# An injectable refrigerated hydrogel for inducing local hypothermia and neuroprotection against traumatic brain injury in mice

**DOI:** 10.1186/s12951-024-02454-z

**Published:** 2024-05-15

**Authors:** Yuhan Han, Zhengzhong Han, Xuyang Huang, Shanshan Li, Guoliang Jin, Junfeng Feng, Decheng Wu, Hongmei Liu

**Affiliations:** 1 Department of Biomedical Engineering, Southern University of Science and Technology, Shenzhen, 518055 Guangdong China; 2 Institute of Nervous System Diseases, Xuzhou Medical University, Xuzhou, 221000 Jiangsu China; 3 Brain Injury Center, Ren Ji Hospital, Shanghai Jiao Tong University School of Medicine, Shanghai Institute of Head Trauma, Shanghai, 200127 China; 4 Department of Neurosurgery, Xuzhou Children’s Hospital, Xuzhou, 221000 Jiangsu China; 5 Department of Intensive Care Medicine, The Second Hospital of Jiaxing, Jiaxing, 314000 Zhejiang China; 6 Department of Forensic Medicine, Xuzhou Medical University, Xuzhou, 221000 Jiangsu China; 7 Department of Neurology, Affiliated Hospital of Xuzhou Medical University, Xuzhou, 221000 Jiangsu China

**Keywords:** Traumatic brain injury, Hydrogel, Local hypothermia, Neuroprotection

## Abstract

**Background:**

Hypothermia is a promising therapy for traumatic brain injury (TBI) in the clinic. However, the neuroprotective outcomes of hypothermia-treated TBI patients in clinical studies are inconsistent due to several severe side effects. Here, an injectable refrigerated hydrogel was designed to deliver 3-iodothyronamine (T1AM) to achieve a longer period of local hypothermia for TBI treatment. Hydrogel has four advantages: (1) It can be injected into injured sites after TBI, where it forms a hydrogel and avoids the side effects of whole-body cooling. (2) Hydrogels can biodegrade and be used for controlled drug release. (3) Released T1AM can induce hypothermia. (4) This hydrogel has increased medical value given its simple operation and ability to achieve timely treatment.

**Methods:**

Pol/T hydrogels were prepared by a low-temperature mixing method and characterized. The effect of the Pol/T hydrogel on traumatic brain injury in mice was studied. The degradation of the hydrogel at the body level was observed with a small animal imager. Brain temperature and body temperature were measured by brain thermometer and body thermometer, respectively. The apoptosis of peripheral nerve cells was detected by immunohistochemical staining. The protective effect of the hydrogels on the blood–brain barrier (BBB) after TBI was evaluated by the Evans blue penetration test. The protective effect of hydrogel on brain edema after injury in mice was detected by Magnetic resonance (MR) in small animals. The enzyme linked immunosorbent assay (ELISA) method was used to measure the levels of inflammatory factors. The effects of behavioral tests on the learning ability and exercise ability of mice after injury were evaluated.

**Results:**

This hydrogel was able to cool the brain to hypothermia for 12 h while maintaining body temperature within the normal range after TBI in mice. More importantly, hypothermia induced by this hydrogel leads to the maintenance of BBB integrity, the prevention of cell death, the reduction of the inflammatory response and brain edema, and the promotion of functional recovery after TBI in mice. This cooling method could be developed as a new approach for hypothermia treatment in TBI patients.

**Conclusion:**

Our study showed that injectable and biodegradable frozen Pol/T hydrogels to induce local hypothermia in TBI mice can be used for the treatment of traumatic brain injury.

**Graphical Abstract:**

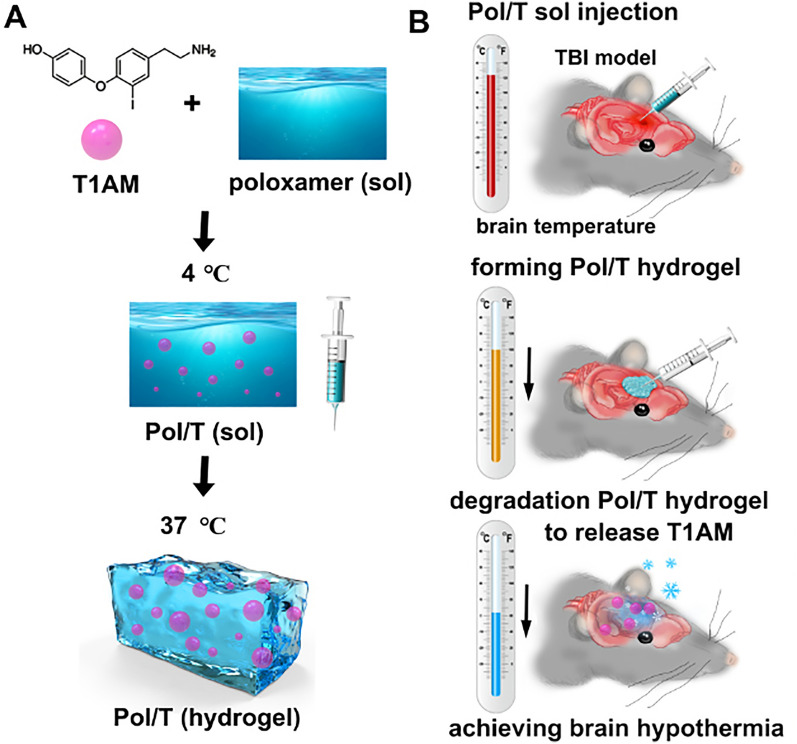

**Supplementary Information:**

The online version contains supplementary material available at 10.1186/s12951-024-02454-z.

## Background

Traumatic brain injury (TBI) is recognized as a leading cause of disability and death worldwide [1–3]. Secondary TBI occurs after the initial mechanism of trauma has progressed [4, 5]. If the initial damage is extensive, secondary TBI occurs, which can result in the initiation of an acute inflammatory response, edema and swelling, continuous breakdown of the blood–brain barrier (BBB), local electrolyte imbalance, and persistent neurological impairment in many patients [6, 7]. In TBI, the activation of various metabolic pathways induced by inflammation and oxidative stress can lead to an excess of neurotoxic metabolic byproducts [8]. Following TBI, oxidative stress triggers the activation of the brain's immune system, resulting in the production of various inflammatory factors and the onset of neuroinflammation. These inflammatory factors also possess neurotoxic properties, creating a toxic environment for neurons through their unrestrained release [9]. Neurotoxicity, neuroinflammation, and oxidative stress are intimately related in the development of TBI, mutually reinforcing each other. Breaking this vicious cycle is crucial for the treatment of TBI. At present, clinical therapeutic agents for TBI that prevent the secondary spread of damage beyond the initial insult are relatively scarce.

Hypothermia exerts strong neuroprotective effects to prevent secondary damage after TBI. The major mechanisms of neuroprotection by hypothermia are attenuation of BBB permeability, reduction of glutamate release, alleviation of neuroinflammation, and reduction of free radical generation and release [10]. In clinical practice, the initial goal in treating patients with TBI is to prevent and correct hypoxemia and hypotension to alleviate secondary injury [11]. Therefore, during the implementation of hypothermia therapy, various measures are taken to ensure the patient’s oxygenation index and circulatory stability [12]. Simultaneously, coagulation dysfunction is promptly corrected, and intracranial pressure is reduced through methods such as osmotherapy and hyperventilation. On the other hand, the use of certain neurotrophic agents can suppress neuroinflammation and improve prognosis [13]. Implementing systemic hypothermia may compromise a patient's oxygenation capacity and circulatory stability, hence the need for safer cooling methods, which is also a significant reason for us to develop new local hypothermia methods. It's worth noting that the neuroprotective outcomes of hypothermia-treated TBI patients have not been consistent across different clinical studies [14, 15]. Two issues limit the therapeutic efficacy of hypothermia: (1) Hypothermia is associated with several severe side effects, such as hypotension, arrhythmias, electrolyte disorders, shivering and local infections, when conventional whole-body cooling is used. In these cases, the adverse effects outweigh the neuroprotective benefits of therapeutic hypothermia [16]. (2) The time of cooling initiation is a key factor that affects the neuroprotective outcomes associated with brain hypothermia. Clinical studies have demonstrated that the window of opportunity for hypothermia therapy is 90 min or less [17, 18]. However, it remains challenging to reduce the time between the injury and induction of hypothermia. It is typically impossible to predict the time spent transporting TBI patients to the hospital. As a result, hypothermia therapy for TBI is largely limited.

To resolve the question of whole-body cooling side effects, targeted brain cooling has been proposed as a reasonable alternative. Local brain hypothermia has the advantage of decreasing the local brain temperature while maintaining whole-body normothermia, potentially achieving the neuroprotective effects of hypothermia with minimal systemic side effects [19, 20]. At present, various methods have been used clinically to achieve local hypothermia, such as epidural placement of a cooling catheter [21], local cold fluid infusion [19], and local ice compression [22], which can have therapeutic effects. However, truly effective and rapid selective brain hypothermia is difficult to achieve, because of the many problems that occur, such as the difficulty and complexity of the surgery, infection of implants, expensive materials, and technical limitations [19, 20, 23]. In addition, it is difficult to solve the time problem when TBI patients begin to experience hypothermia. Therefore, the discovery of an effective and timely approach to achieve local brain hypothermia remains challenging.

Injectable hydrogels can be used for the local delivery of drugs and have been widely used for the treatment of brain diseases (TBI and stroke) due to their good biocompatibility, biofunctionality, and ability to form via an in situ gelation process [24, 25]. Hydrogels themselves can be used as carriers to load various drugs, and the drug utilization efficiency can be improved by prolonging the action time of the drug by injecting it in situ or directly on the tissue surface [23]. Moreover, these hydrogels are easy to carry and inject into wounds in a timely manner. Many studies have shown that these hydrogels are more advantageous for treating many diseases, such as spinal injury, stroke and glioma than traditional therapies [26–28]. SURGIFLO® (a gelatin-based hydrogel) and the Porcine Fibrin Sealant Kit (a collagen-based hydrogel) have been widely used for hemostasis and prevention of cerebrospinal fluid leakage after neurosurgery [29, 30]. Therefore, the clinical application of hydrogels appears promising. Inspired by these hydrogels, we hypothesized that a hydrogel could be developed to achieve hypothermia.

3-iodothyronamine (T1AM) is an endogenous derivative of thyroid hormones. Many studies have reported that T1AM can interact with specific receptors and produce profound hypothermia within minutes in vivo after systemic administration [31, 32]. T1AM can penetrate the BBB and is detected in the brain. T1AM has been used in animal models to protect against stroke-induced brain injury and is able to provide marked neuroprotection [33]. However, T1AM administration by intraperitoneal injection is associated with some side effects because the binding sites of T1AM are widely distributed in various organs, such as the heart, aorta, kidney, stomach, and small intestine [34–36]. Furthermore, the rapid metabolism of T1AM in vivo limits its effective concentration in the brain [37, 38]. Long-term systemic administration and high doses can cause side effects, such as hypotension, heart failure, hyperglycemia, ketoacidosis, a decline in kidney function and muscle weakness [35, 36, 39]. Therefore, there is an urgent need to identify more effective methods to reduce the systemic distribution and systemic clearance of T1AM.

**Scheme 1:**
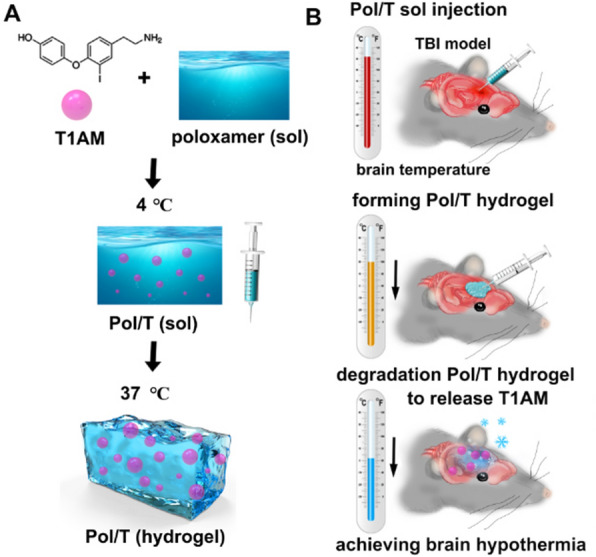
Formation and mechanism of the Pol/T hydrogel in traumatic brain injury (TBI) mice. **A** Schematic illustration of Pol/T hydrogel preparation procedures and sol–gel transition properties at 37 °C. **B** In situ postsurgical injection of Pol/T sol into the brain after TBI; the Pol/T hydrogel was formed and biodegraded in the brain trauma microenvironment (BTE) to release 3-iodothyronamine (T1AM) to achieve mouse brain hypothermia

The advantages of hydrogels allow us to believe that we can use them as drug carriers to achieve timely and long-lasting effective local hypothermia to avoid multiple problems with existing methods. In this study, we used an injectable poloxamer (Pol) hydrogel to deliver T1AM (Pol/T hydrogel) to achieve timely and effective local hypothermia for TBI mouse treatment (Scheme 1). After the Pol/T hydrogel was formed, the Pol hydrogel biodegraded in the mouse brain trauma microenvironment (BTE); thus, T1AM was released for hypothermia treatment. Importantly, we used this hydrogel to deliver T1AM directly to the injured mouse brain area, effectively achieving brain hypothermia and avoiding the side effects of systemic medication in other organs. Given its ease of preparation, injectability, and local drug delivery, this strategy using a Pol/T refrigerated hydrogel induced local cerebral hypothermia and showed great potential for neuroprotection against TBI.

## Methods

### Preparation of the Pol hydrogel and Pol/T hydrogel

0.25 g of Pol 407 (25% w/v) and 0.5 g of Pol 188 (5% w/v) were added to 1 mL of high-purity water and placed in a refrigerator at 4 °C until they were fully dissolved. The sol-to-pol transition at physiological temperature was measured at 37 °C in a water bath. For the preparation of the Pol hydrogel, 0.25 g of Pol 407 (25% w/v) and 0.5 g of Pol 188 (5% w/v) containing 50 μg of T1AM were mixed in 1 mL of high-purity water and stored at 4 °C until use.

### Rheological monitoring of the Pol/T hydrogel

Dynamic rheometry measurements were carried out on a dynamic Discovery HR-2 rheometer (TA Instruments, USA) equipped with electrically heated plates. Hydrogel samples (400 µL) were added to a parallel plate (diameter 40 mm) using a syringe, and the gap betweent the plate was set to 500 μm. The storage modulus (Gʹ) and loss modulus (G″) data were recorded at 4–45 °C using a strain control of 5%.

### Micromorphology of the Pol/T hydrogel

The Pol/T hydrogels were frozen to a solid state in liquid nitrogen, and then the samples were critically dried via vacuum freeze dryer for 48 h*.* The freeze-dried samples were fixed on aluminium plates. After the powder was sputter-coated with gold, the surface morphology of the Pol/T hydrogel was observed by SEM (FEI Teneo VS).

### Degradation studies in vivo

For the assessment of Pol hydrogel degradation in vivo, Pol hydrogels (50 µL) loaded with DiR were injected into weight-drop injury (WDI) model mice, and those mice were imaged with a Xenogen IVIS Spectrum optical device (Caliper, USA) to measure the fluorescence intensity of 1,1ʹ-dioctadecyl-3,3,3ʹ,3ʹ-tetramethylindotricarbocyanine iodide (DiR) at 3, 6, and 12 h. The data are presented as the means ± SDs (n = 3 mice).

### Patients and CSF samples

The human study was approved by the Ethics Committee of the Affiliated Hospital of Xuzhou Medical University (XYFY2020-KL208-01). All patients provided written informed consent, and all specimens were handled and anonymized according to ethical and legal standards. CSF samples were collected from TBI patients at the Affiliated Hospital of Xuzhou Medical University by lumbar puncture.

### In vitro and in vivo drug-release from Pol/T hydrogel

To measure the release of T1AM from the Pol/T hydrogels, 500 µL of each Pol/T hydrogel was transferred to a 4 mL centrifuge tube and suspended in 3 mL of cerebrospinal fluid (CSF). The samples were incubated at 37 °C, and the T1AM released from the Pol/T hydrogels was quantified at different time intervals by high-performance liquid chromatography (Waters e2695) and mass spectrometry (Waters UPLC-ESI-TQD). The column was an advanced Hypersil C18 column (250 × 4.6 mm), and the 1 mL/min mobile phase consisted of methanol: water (45:55) with 0.01% trifluoroacetic acid. The mass spectrometer parameters were as follows: capillary, 0.6 kV; cone, 20 V; collision, 20 V; ion source temperature, 150 °C; gas flow, 800 L/h; and cone gas flow, 20 L/h56. The transitions used for T1AM identification and quantification were 355.904–194.5861 and 355.904–212.0184, respectively. The data are presented as the means ± SDs (n = 3 mice/time).

To measure the T1AM content in the tissues, the cerebral hemispheres and hypothalamus on the traumatized side of the mice treated with phosphate buffer saline (PBS) and Pol/T were dissected at 3 h, 6 h and 12 h. The cerebral hemisphere and hypothalamus tissue samples were homogenized and sonicated for 60 min at 4 °C under dark conditions. Then, 800 μL of methanol was added to the tissue samples to precipitate the proteins. The above mixed tissue samples were vortex mixed for 10 min and centrifuged at 13,000 rpm at 4 °C for 5 min. Then, 100 μL of the supernatant was taken for measurement. The detection method was as described above.

### TBI animal models

All experimental procedures were approved by the Xuzhou Medical University of China Animal Care and Use Committee. Five-week-old ICR male and female mice were purchased from Beijing HFK Bioscience Co., Ltd. (Beijing, China). The experimental animals were kept isolated from each other in a cage with an independent air supply. The animals were housed 5 per cage before TBI and then housed singly afterward. After receipt from the vendor, the mice were allowed to acclimate for 1 week. The room temperature was controlled at 23 ± 2 °C. Regular light was maintained (12 h day, 12 h night), and a sufficient supply of water and food was maintained. All mice were healthy at the start of the TBI procedure. A small animal anesthesia machine was used, and isoflurane was used for anesthesia. Anesthesia in mice deepens into the surgical stage: respiratory rate decreases, abdominal breathing becomes predominant, muscles fully relax, eyelid reflex disappears, corneal reflex weakens, and there’s no response to toe or finger pinch. The steps for establishing the WDI models were as follows. A 5 mm craniotomy (3 mm posterior and 3 mm lateral from bregma) was performed on the right parietal region. A weight-drop-hitting device (ZH-ZYQ, Anhui Zhenghua Biological Instrument Equipment Co., Ltd., Huaibei, China) with a 4.0 mm diameter footplate was used to induce injury. Forces of impact were produced by a 40 g weight drop from a height of 7.5 cm. A model of the penetrating brain injury (PBI) was established as follows. At the same position, 9 needles were punctured according to a 3 × 3 grid, with a depth of 3 mm, a width of 1 mm and a height of 1 mm.

The Pol hydrogel group and Pol/T hydrogel group were injected with 50 μL hydrogel, and the T1AM group was injected intraperitoneally with 50 mg/kg of T1AM after trauma. The TBI control group did not receive any therapy. The mice in the rewarming group were immediately rewarmed after being injected with 50 μL of Pol/T hydrogel to maintain the brain temperature at 36 °C by heating the probe through the hollow coil for 12 h (Additional file 1: Figure S1). To reduce the effects of the anesthetic on the rewarmed group, the rewarmed group was heated in the light to maintain a normal body temperature. A temperature probe was inserted into the rectum to monitor the body temperature, and the shivering response of the mice was observed. These measures ensured that the brain and body temperature of the mice in the rewarmed group remained constant throughout the procedure and offset the hypothermia caused by anesthesia. The delayed group was injected with 50 μL of Pol/T hydrogel at 6 h postinjury. The sham group underwent right parietal craniotomy but did not undergo any injury. The sham + Pol group or sham + Pol/T group underwent right parietal craniotomy and were injected with 50 μL of Pol or Pol/T hydrogel.

### Temperature measurement

Five-week-old male and female ICR mice were used. TBI was induced via the WDI or PBI, and the treatments were administered to the mice individually. Mice were carefully monitored 2 h after TBI surgery in the cages for waking. If a mouse died after TBI but before the end of the 2 h observation period (2 h after TBI), the data were not included in the analysis. Twenty male mice were equally randomized among these 5 groups: (i) Sham, (ii) Control, (iii) T1AM, (iv) Pol, and (v) Pol/T after the administration of TBI. For every 4 contiguous TBI administrations, one mouse was selected from each of the 5 groups. The order in which the mice were selected from the 5 groups was the same, but the starting group changed with each set of 5 mice. For example, for the first set of 5 mice, the group order was i, ii, iii, iv, and v; for the next set of 5 mice, the order was ii, iii, iv, i, and v. Two animal technicians administered the TBI and subsequent treatments. Three animal technicians collected the data from the mice during the 12 h observation period. Two of these three patients also underwent TBI and treatment and thus were not blinded to treatment when the data were recorded; the one who had not participated in the TBI treatment was blinded. The statistical analyst was not blinded to the groups. The data from 4 mice/group (20 total) were analyzed.

The body and brain temperatures of the mice were measured with probes (Omega Engineering, China). Rectal temperature was measured to represent body temperature. To measure the brain temperature of the mice, a hollow coil was fixed on the surface of the cerebral cortex during modeling. The coil was placed in the bone window and attached to the cerebral cortex, and the coil was fixed with a silk thread. The mice in the rewarming group were continuously anesthetized, and a custom-made oropharyngeal airway was used to maintain patchy breathing. The brain was continuously rewarmed by a heating probe through a hollow coil, and the body of the mice was heated by light so that the body temperature of the mice remained normal. Except for the rewarming group, each mouse was artificially fixed without anesthesia each hour, and the temperature probe was measured through a hollow coil fixed on the dural membrane. The brain temperature was measured along with the anal temperature, which was used to represent the body temperature of the mice. All the mice were measured at 1 h intervals for 12 consecutive hours. These data were obtained by a temperature acquisition system controlled by an instrument (Omega Engineering, China).

### Vital sign measurement

The experimental animals, sample size per group, randomization, inclusion and exclusion criteria and blinding methods used were the same or similar to those used for the temperature measurements. Differences are noted. Twenty male mice were equally randomized among these 5 groups: (i) Sham, (ii) Control, (iii) T1AM, (iv) Pol, and (v) Pol/T after the administration of TBI. Vital signs, including blood pressure (mm/Hg), heart rate (beats/min), respiratory rate (beats/min) and oxygen saturation (%), were recorded by a life sign monitor (Anhui Zhenghua Biological Instrument Equipment Co., Ltd., Huaibei, China). All the mice were measured at 1-h intervals for 12 consecutive hours. Four mice were measured in each group. In the process of measurement, except for the rewarming group, which was continuously anesthetized, the mice were artificially fixed without anesthesia, and the electrodes were connected to the mice. Life sign monitors automatically displayed the relevant vital sign values.

### Spectrophotometric assay for hemoglobin content

The experimental animals, sample size per group, randomization, inclusion and exclusion criteria and blinding methods used were the same or similar to those used for the temperature measurements. Differences are noted. Twenty-eight male mice were equally randomized among these 7 groups: (i) Sham, (ii) Control, (iii) T1AM, (iv) Pol, (v) Pol/T, (vi) Delay, and (vii) Rewarming after the administration of TBI. The hemoglobin content of brains subjected to traumatic injury and subjected to different treatments was quantified using a spectrophotometric assay with some modifications [40]. At 12 h after TBI, the mice were deeply anesthetized with isoflurane. The mice were perfused transcardially with PBS. Then, the brain was removed from the skull. The sides of the traumatic lateral hemisphere of the brain were separated, weighed and placed into 4 mL EP tubes. Brain tissue was homogenized on ice and sonicated for 1 min. Brain tissue homogenates were centrifuged at 12,000 r/min for 30 min at 4 °C. A standard curve of hemoglobin was constructed in a 96-well plate that contained increasing ratios of hemoglobin standard to Drabkin reagent. The supernatant of the brain tissue homogenate was added to Drabkin reagent and incubated for 15 min for spectrophotometric analysis.

### Mouse tissue immunohistochemistry (IHC) staining

The experimental animals, sample size per group, randomization, inclusion and exclusion criteria and blinding methods used were the same or similar to those used for the temperature measurements. Differences are noted. Mouse brain tissues were fixed in 4% paraformaldehyde in sodium citrate buffer and embedded in paraffin for histological analysis. The data from 3 mice/group were analyzed. Hematoxylin and eosin (H&E) staining were performed on 20 µm sections cut with a paraffin slicing machine.

Apoptotic cells in brain tissues were detected by a Tunel BOSTER (MK014) according to the manufacturer's protocol. In brief, brain tissue sections were incubated with 20 µg/mL protease K and rinsed with 0.3% Triton X-100. The TdT-mediated dUTP nick-end labeling (TUNEL) reaction mixture was added to the brain tissue sections for 2 h at 37 °C in a humidified atmosphere in the dark. Apoptosis was measured by diaminobenzidine (DAB) staining.

Paraffin-embedded brain tissue sections were deparaffinized and boiled in sodium citrate buffer. Then, the brain sections were incubated with normal goat serum. After that, each section was incubated with different primary antibodies against B-cell lymphoma-2 (Bcl-2) (Abcam, ab32124), BCL2-Associated X (Bax) (Abcam, ab81083), matrix metalloproteinase-9 (MMP9) (Abcam, ab236494), ionized calcium binding adapter molecule 1 (Iba-1) (Sigma, SAB2702364), glial fibrillary acidic protein (GFAP) (Cell Signaling Technology, 12389 s) at 4 °C for 12 h. The first antibody was diluted to different concentrations as follow: Bcl-2 (1:100), Bax (1:500), MMP9 (1:100), GFAP (1:200), Iba-1 (1:500). Then, the sections were incubated with a goat anti-rabbit secondary antibody (ZSBio, Beijing, China) for 40 min. Finally, each section was stained with DAB. After 3 washes in PBS and mounting with glycerin, a Leica microscope was used for observation of tissue sections, and the number of positive cells was counted. During the counting process, we based cell existence on the presence of the cell nucleus. Only when positive protein expression co-localized with the cell nucleus was it counted as a positive cell. Five fields of nonoverlapping areas around the wound were taken from each section, and 3 mice were included in each group.

### Measurement of cerebral edema and Evans blue (EB) extravasation study

The experimental animals, sample size per group, randomization, inclusion and exclusion criteria and blinding methods used were the same or similar to those used for the temperature measurements. Differences are noted. The data from 5 mice/group were analyzed. Cerebral edema was determined by measuring the brain water content at 12 h post-TBI. Following anesthesia by isoflurane and decapitation, the mice were sacrificed, and the brains were collected immediately. The ipsilateral percussion side was separated and weighed to obtain the wet weight. Then, the brain specimens were dried in a desiccating oven at 80 °C for 72 h and weighed again to determine their dry weight. The brain water content was calculated using the following formula: (wet weight-dry weight)/(wet weight). BBB permeability was measured by evaluating the extravasation of EB at 12 h post trauma. EB dye (2%, 2 mL/kg) was administered intravenously at 9 h after TBI and then allowed to circulate for 3 h to ensure that the EB dye fully circulated through the blood system. After anesthesia, 100 mL of PBS was perfused through the left ventricle of the heart, and the brain was removed. The ipsilateral percussion side was separated and weighed. Then, the tissue was placed in 4 mL of potassium hydroxide (1 M) and homogenized. The 1 mL mixture was blended with 5 mL of 0.2 M phosphoric acid and acetone (5:12) and centrifuged at 3000 rpm for 30 min. The supernatant was transferred, and the absorbance at 620 nm was detected.

### Magnetic resonance imaging examination

Experimental animals, sample size per group, randomization, inclusion and exclusion criteria and blinding were the same or similar as in experiment of temperature measurement. Differences are noted. Data from 5 mice/group were analyzed. On the 12th h after the TBI, T2-weighted sequence and diffusion-weighted imaging (DWI) was evaluated by small animal-specific 7 T Micro-MR (Bruker, Bio Spin MRI Pharma Scan 7.0 T). The mice were mildly anesthetized with isoflurane (3.5% induction, and 1.5% maintenance). Throughout the process, the life sign monitors continuously monitored vital signs. According to the vital signs, the inhalation flow was adjusted in time to avoid the death of mice. Once death occurred, the experiment was excluded. The T2-weighted MRI parameters were: repetition time: 2500 ms, echo time: 36 ms, slice thickness: 1 mm, field of view: 20 × 20 mm, image matrix: 256 × 256. The DWI parameters were: repetition time: 3000 ms, echo time: 20 ms, slice thickness: 1 mm, field of view: 100 × 110 mm. All MRI were evaluated by a proficient neuroimaging doctor who was blinded to the study design. Based on the T2 images, the area of edema was measured using Image-J software. The ADC value size was automatically calculated by the Paravision 6.1 of the MRI scanner based on the DWI and the DWI image was processed by the Paravision 6.1 with pseudo-color.

### Enzyme linked immunosorbent assay (ELISA)

The experimental animals, sample size per group, randomization, inclusion and exclusion criteria and blinding methods used were the same or similar to those used for the temperature measurements. Differences are noted. The data from 3 mice/group were analyzed. The mice were anesthetized and euthanized, and the brains were then removed at 12 h after injury. Then, the ipsilateral percussion side was separated and weighed. The tissue was fully homogenized in lysis solution and centrifuged at 12,000 rpm for 10 min. The supernatant was assessed by ELISA kits (Boster Biological Technology, Wuhan, China) according to the manufacturer’s instructions.

### Neurologic function assessment

The experimental animals, sample size per group, randomization, inclusion and exclusion criteria and blinding methods used were the same or similar to those used for the temperature measurements. Differences were noted. The data from 5 mice/group were analyzed. The Morris water maze experiment was used to evaluate the recovery of spatial memory and learning ability in mice. All mice were tested for 6 days from the 21st day to the 26th day after TBI. During the first 5 d, the mice were randomly placed in the water in a certain quadrant, and the mice could swim freely in the pool until they found the platform to rest. On the 6th day, the platform was removed, and the platform quadrant was randomly selected. All the data collected during the test were recorded and analyzed by a video tracking system (Anhui Zhenghua Biological Instrument Equipment Co., Ltd., Huaibei, China). The residual motor dysfunction was evaluated by the modified neurological severity score (mNSS) and the wire hanging test at 21 d after TBI. The mNSS included rotation, cylinder, corner, and beam tests. In the course of the wire hanging test, the mice were placed on metallic wire, and the wire was inverted. The latency to fall was recorded.

### Statistical analyses

All the statistical analyses were performed using SPSS version 16.0, and the statistical graphs were generated with GraphPad Prism 8.0. If the quantitative data conformed to a normal distribution, the data are presented as the means ± SDs. If the quantitative data did not conform to a normal distribution, the data are presented as quartiles (Q50 (Q25, Q75)). Qualitative data are expressed as percentages or constituent ratios. If multiple sets of quantitative data conformed to a normal distribution and homogeneity, statistical significance was analyzed using one-way analysis of variance (ANOVA) with Tukey’s post hoc test and repeated measures one-way ANOVA with post hoc Bonferroni analysis. If multiple sets of quantitative data did not conform to a normal distribution or homogeneity, the statistical significance of differences between multiple groups was analyzed using the Kruskal‒Wallis test.

## Results

### Characterization of the hydrogel

**Fig. 1:**
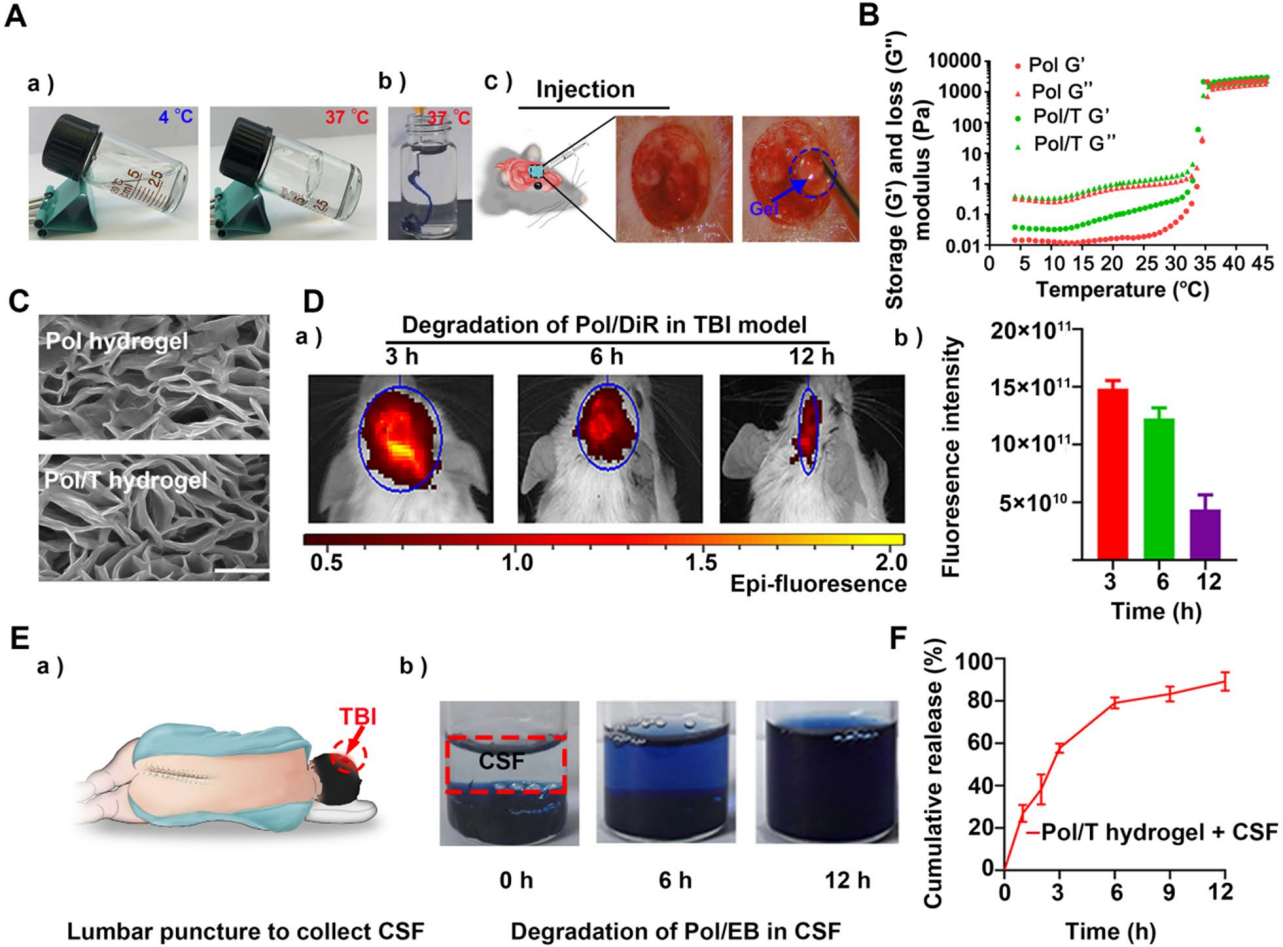
Preparation and characteristics of the Pol/T hydrogel. **A** Gelation and syringe ability of the Pol/T hydrogel at 37 ℃ in vitro (a and b). The Pol/T hydrogels were injected into the postsurgical cavity of TBI mice, and the Pol/T hydrogels formed a hydrogel in vivo (c). **B** Storage (Gʹ) and loss (G″) moduli of Pol and Pol/T as a function of temperature from 4 to 45 °C. **C** SEM images of the Pol hydrogels and the Pol/T hydrogels. **D** In vivo release kinetics of 1,1ʹ-dioctadecyl-3,3,3ʹ,3ʹ-tetramethylindotricarbocyanine iodide (DiR) from the Pol/DiR hydrogel in response to the posttraumatic environment (a) and quantification of the DiR fluorescence signal (b). The data are presented as the means ± SDs (n = 3 mice). **E** Cerebrospinal fluid (CSF) was collected from clinical TBI patients by lumbar puncture (a), and the Pol/EB hydrogels were degraded in the CSF from TBI patients (b). **F** In vitro release kinetics of T1AM from the Pol/T hydrogels in the CSF of a TBI patient. The data are presented as the means ± SDs (n = 3 mice/time)

Pol 407 and Pol 188 were mixed at different mass ratios to form hydrogels, which are liquid at 4 °C and transform into gels at body temperature (36–37 °C). As shown in Fig. 1Aa, when the mixture was composed of 25% w/v high Pol 407 and 5% w/v low Pol, the prepared hydrogel could transform into a gel at 37 °C. To clearly observe the process of Pol at 37 °C, EB dye was added to the Pol hydrogel to generate a blue Pol/EB. Sol Pol/EB (4 °C) was injected into 37 °C water, after which the Pol/EB hydrogel quickly formed (Fig. 1Ab). Furthermore, we investigated the sol–gel transition and injection behavior of the Pol hydrogel in vivo using a TBI model of Feeney’s WDI. The sol pol could transform into a gel pol after 2 min in the wound cavity of a TBI mouse (Fig. 1Ac). Rheology tests revealed that the Pol and Pol/T hydrogels displayed similar sol-to-gel transitions at approximately 35 °C, suggesting that Pol and Pol/T could form hydrogels at body temperature (Fig. 1B). The porous structure of the Pol hydrogel was observed by scanning electron microscopy (SEM). T1AM at 50 mg/mL within the Pol hydrogel did not affect the structure of the hydrogel (Fig. 1C). To test the biodegradation property of this Pol/T hydrogel in vivo, DiR was embedded into the Pol hydrogel (Pol/DiR). As shown in Fig. 1Da, b, the fluorescence signal decayed, and the lowest fluorescence intensity was observed 12 h after injection, suggesting that the Pol hydrogel could biodegrade in the BTE over the 12-h period.

To further monitor the degradation behavior of the Pol hydrogel in an environment similar to that of the human TBI environment, we collected CSF from clinical TBI patients and soaked the hydrogel in CSF to verify the biodegradation behavior of Pol in vitro (Fig. 1Ea). Pol/EB was mostly biodegraded by 12 h (Fig. 1Eb), and approximately 85% of T1AM was released from the Pol hydrogel during incubation in CSF within 12 h (Fig. 1F). The concentration of T1AM in the cerebral hemisphere on the injured side was detected after injection of the Pol/T hydrogel. As shown in Additional file 1: Figure S2, T1AM (13.85 ± 10.17 μmol/g) was detected in the injured cerebral hemisphere 12 h after injection of the Pol/T hydrogel. Taken together, these results demonstrate that the Pol hydrogel could biodegrade and accomplish the controlled release of T1AM in a posttraumatic environment.

The safety of materials is the primary problem in clinical applications. Pol hydrogels are polymers with good biological safety and are approved by the FDA. In this study, the cytotoxicity of the Pol hydrogel was determined by the MTT assay. As shown in Additional file 1: Figure S3, the survival rate of the cells was greater than 90% after they were incubated for 3, 6, or 12 h in the Pol hydrogel immersion solution, which indicates that the Pol hydrogel exhibited highly favorable safety.

### Local hypothermia was induced by the Pol/T hydrogel without leading to systemic negative effects

**Fig. 2:**
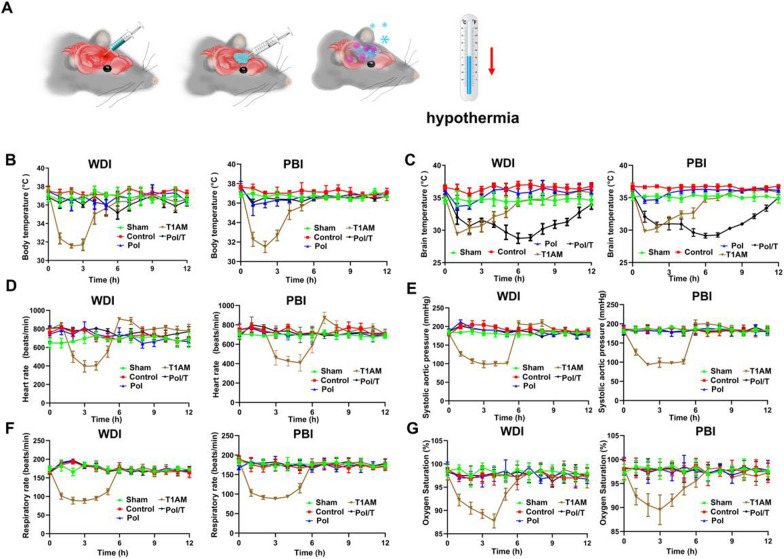
The Pol/T hydrogel provided effective and long-term local hypothermia and avoided systemic side effects that occur as a result of whole-body hypothermia. **A** Diagram of local hypothermia via the Pol/T hydrogels. **B** Changes in the body temperature of male TBI mice over time after the mice received different treatments. **C** Changes in the temperature of the brain of male TBI mice over time after they received different treatments. **D** The variation in heart rate with time in the different groups. **E** The variation in systolic blood pressure with time in the different groups. **F** The variation in respiratory rate with time in the different groups. **G** The variation in oxygen saturation with time in the different groups. **B**–**G** The data are presented as the means ± SDs (n = 4 mice). Statistical significance was determined using repeated measures one-way ANOVA with a post hoc Bonferroni correction

To test the curative effect of the Pol/T hydrogel, WDI and PBI models were generated in this study. Local administration of free T1AM via a hydrogel to the mouse brain would achieve local hypothermia to reduce side effects caused by whole-body cooling (Fig. 2A). To investigate the effect of hypothermia induced by the Pol/T hydrogel, the concentration of T1AM in the thermoregulatory center of the hypothalamus was detected. In this study, we measured the concentration of T1AM in the hypothalamus of mice treated with PBS or the Pol/T hydrogel. The concentration of T1AM in the hypothalamus of mice in the different groups did not differ by more than 1.41 pmol/g (Additional file 1: Figure S4). This result suggested that local administration of the Pol/T hydrogel prevents systemic cooling caused by the hypothalamus.

Then, we assessed the temperature variation in the mouse brain and body. The rectal temperature was representative of the body temperature. The safety of the doses of free T1AM and T1AM (from 50 to 100 mg/kg) was assessed based on the survival curves of the mice after intraperitoneal injection. As shown in Additional file 1: Figure S5, all five mice survived for 12 h after treatment with 50 mg/kg T1AM (consistent with a report by Thomas S Scanlan) [31], and some mice died when treated with concentrations greater than 50 mg/kg T1AM. When the dose of T1AM was 100 mg/kg, the mice became inactive, assumed a slightly hunched-back posture and developed ptosis (drooping eyelids), and some mice even died within 1 h of intraperitoneal injection (see Additional file 2: Movie S1). Nevertheless, when we used Pol as a T1AM carrier to deliver 100 mg/kg T1AM directly to the brain, treated mice exhibited behaviors similar to those of normal mice, and the side effects of whole-body cooling were avoided (see Additional file 3: Movie S2). According to our experimental results and data reported by Thomas S Scanlan [31], 50 mg/kg T1AM was used for all subsequent experiments.

Body and brain temperatures were measured after treatment with Pol, free T1AM and Pol/T at all time points up to 12 h in adult male mice in the WDI and PBI models (Fig. 2B, C). The brain temperature of TBI mice injected with the Pol hydrogel decreased by approximately 2.0–3.9 °C from the control brain temperature for less than 2 h, whereas the body temperature of the treated Pol hydrogel differed by more than 2.8 °C. These findings suggested that the conversion of Pol hydrogel from liquid (4 °C) to gel (37 °C) decreases brain temperature. Intraperitoneal injection of free T1AM rapidly reduced the whole-body temperature (including body and brain temperature), and this low temperature was maintained for only approximately 4 h in both the brain and body of TBI mice due to systemic effects and the short-acting nature of T1AM. Compared with those in the Pol and free T1AM treatment groups, the brain temperature of the male TBI mice treated with the Pol/T hydrogel slowly decreased from normal temperature to low temperature (WDI: 28.75 ± 0.79 °C; PBI: 29.15 ± 0.37 °C) within 6 h and then slowly returned to a normal temperature from 6 to 12 h after injection. As shown in Additional file 1: Figure S6, the body and brain temperatures of male and female mice treated with the Pol/T hydrogel in the WDI model were detected at each time point, and there was no difference between the body and brain temperatures of male and female mice. The maximum difference in body temperature between the sexes was 2.1 °C, and the maximum difference in brain temperature between the sexes was 2.8 °C. The above results suggested that the brain temperature and body temperature tended to differ between male and female mice after treatment with the Pol/T hydrogel. Therefore, male mice were used in the following experiments. The brain temperature of sham group mice treated with Pol and Pol/T was measured. The results showed that the brain temperature of the sham group treated with the Pol/T hydrogel exhibited hypothermia for 12 h, which was consistent with what was observed in the Pol/T hydrogel-treated TBI mice (Additional file 1: Figure S7). The above results suggest that T1AM embedded in the Pol hydrogel was released slowly over time to maintain a cool brain temperature. The negative systemic effect of whole-body hypothermia limits its clinical application, but local hypothermia can effectively prevent this negative effect. In this study, heart rate, systolic blood pressure, respiratory rate and oxygen saturation were monitored for 12 h after the trauma. Free T1AM has already been confirmed to have a negative effect on the heart and causes a decrease in cardiac output [31]. Similarly, the heart rate and systolic blood pressure of free T1AM-treated TBI mice decreased within 6 h compared with those of sham group mice (Fig. 2D and E). As the temperature returned to normal, the heart rate and systolic blood pressure suddenly increased and even exhibited a rebound effect that increased these two vital signs in free T1AM-treated mice. Similarly, the respiratory rate and oxygen saturation of free T1AM-treated TBI mice were reduced within 6 h compared with those of sham group mice (Fig. 2F and G). Then, we detected these vital signs in TBI mice in the Pol/T group. The heart rate did not fluctuate by more than 106 beats/min in the WDI models or 118 beats/min in the PBI models. The systolic blood pressure did not fluctuate by more than 47 mmHg in the WDI models or by 37 mmHg in the PBI models. The respiratory rate did not fluctuate by more than 50 beats/min in the WDI models or by 40 beats/min in the PBI models. The oxygen saturation did not fluctuate by more than 5% in the WDI models or by 5% in the PBI models (Fig. 2D–G). These results showed that these vital signs did not fluctuate appreciably when we used the Pol/T hydrogel to induce local hypothermia in the brain. Hypothermia can aggravate bleeding, but this is not necessarily always the case. Spectrophotometric hemoglobin was used to measure blood content as described in previous work [40]. Additional file 1: Figure S8 shows that there was no significant difference in the hemoglobin content among the TBI control, pol, pol/T, delay and rewarming groups (P > 0.05). However, the hemoglobin content of the T1AM group (WDI: 51.74 ± 1.48 µL, PBI: 57.55 ± 1.03 µL) was the highest among all the groups, indicating that whole-body hypothermia tended to induce bleeding. The results suggested that the local hypothermia induced by the Pol/T hydrogel effectively avoided the risk of bleeding from whole-body hypothermia. To verify the impact of Pol and Pol/T on normal brain tissue subjected to moderate to deep cooling, IHC was used to test the expression of TUNEL, Bcl-2, Bax and MMP9. As shown in Additional file 1: Figure S9A and B, Pol/T’s mean was not more than 2.131, 4.839, 2.545 or 2.501 units, which was greater than the mean for TUNEL-, Bcl-2-, Bax- or MMP9-positive cells in the Sham group, respectively, suggesting that there were no negative effects on brain tissue. These phenomena showed that the Pol/T hydrogel induced a low temperature in the brain, but this reduction in temperature did not negatively affect the mice. The above results demonstrated that the Pol/T hydrogel could induce effective and timely local hypothermia after TBI without side effects.

### Effect of local hypothermia induced by the Pol/T hydrogel on trauma-induced neuronal injury

Many studies have reported that therapeutic hypothermia has a long-term neuroprotective effect after TBI [41–43]. To assess the lesion volume of the brain 21 d post-TBI, serial coronal brain sections were prepared and stained with H&E. As shown in Additional file 1: Figure S10, Pol/T hydrogel-treated TBI alleviated parenchymal tissue loss after TBI. To further validate the neuroprotective effect of the Pol/T hydrogel, neuronal apoptosis and necrosis following TBI were tested. Compared with those in the sham group, there were more TUNEL-positive cells in the TBI control group, which demonstrated substantial overall cell death posttrauma (Additional file 1: Figure S11A and B). We observed that the overall number of TUNEL-positive cells in Pol/T mice was lower than that in free T1AM mice for the WDI and PBI models because the Pol/T hydrogel induced local hypothermia for 12 h. Compared with the rewarming and delayed hypothermia groups, the Pol/T hydrogel-treated TBI mice had the smallest number of TUNEL-positive cells, suggesting that timely and longer local hypothermia of the Pol/T hydrogel could achieve the best neuroprotective effect (Additional file 1: Figure S13A and B).

**Fig. 3:**
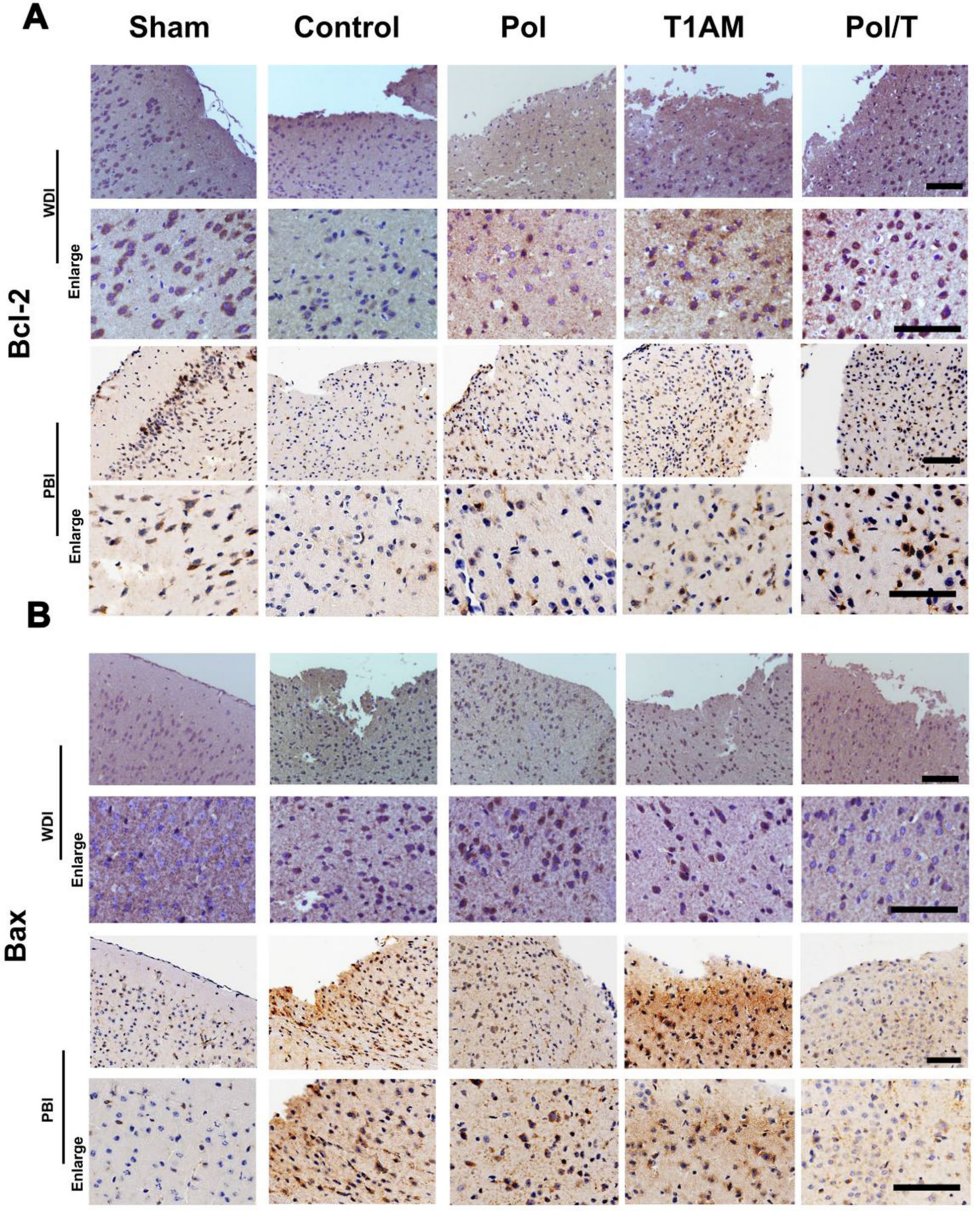
The Pol/T hydrogel-induced hypothermia reduced tissue damage. **A** Representative fields of B-cell lymphoma-2 (Bcl-2) staining around the injury site in the different groups. **B** Representative fields of BCL2-Associated X (Bax) staining around the injury site in different groups (b). Scale bar = 100 μm

Bcl-2 and Bax are antagonistic factors. Bcl-2/Bax regulates mitochondrial function and the release of apoptosis-related proteins [44]. We used IHC to determine Bcl-2 and Bax expression at the edge of the injured area. Bcl-2 expression was greater in Pol/T hydrogel-treated mice than in mice subjected to other treatments. On the other hand, Bax expression decreased compared to that in mice administered other treatments (Fig. 3 and Additional file 1: Figure S13C). The ratio of anti-apoptosis-related Bcl-2/Bax in the brain tissue of the mice subjected to Pol/T hydrogel treatment showed that differences may be evident among the groups subjected to TBI (Additional file 1: Figure S12). Compared with those in the rewarming and delayed hypothermia groups, the ratio of anti-apoptosis-related Bcl-2/Bax in the Pol/T hydrogel-treated TBI group increased (Additional file 1: Figure S13D). The above results indicate that the Pol/T hydrogels could inhibit Bax expression and upregulate Bcl-2 expression after TBI. Taken together, these results suggested that the Pol/T hydrogel induced timely and longer local hypothermia and protected neurons from damage caused by TBI.

### Effect of local hypothermia induced by the Pol/T hydrogel on BBB permeability and brain edema after TBI

**Fig. 4:**
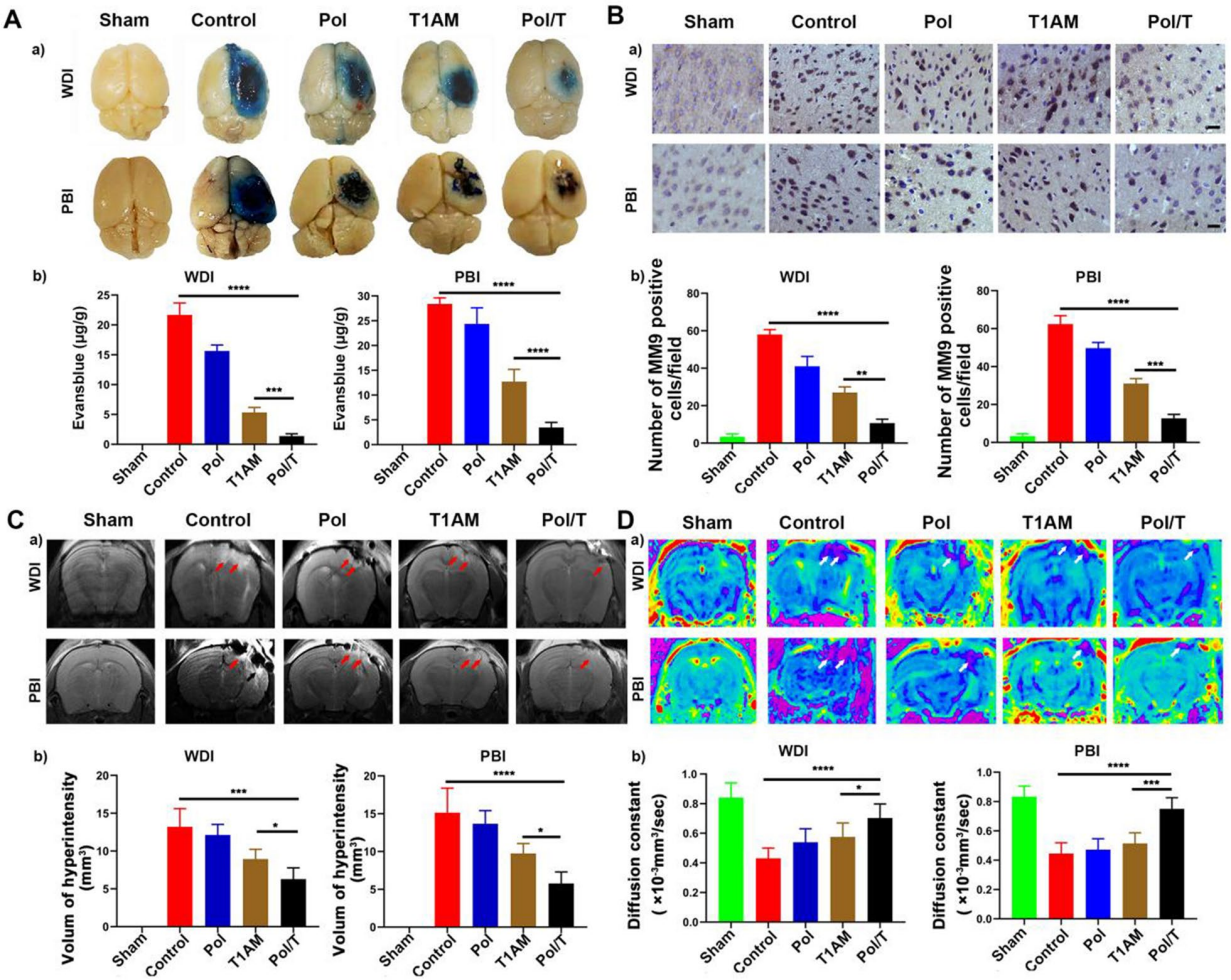
Local hypothermia induced by the Pol/T hydrogel protected the integrity of the BBB and reduced brain edema in the TBI mouse model. **A** Images of EB leakage from brain capillary vessels in the right brain of mice in each group at 12 h (a). Quantification of Evans blue (EB) leakage in each group at 12 h (b). **B** Expression of matrix metalloproteinase-9 (MMP9) in the injured tissue at 12 h. Scale bar = 50 μm (a). Number of MMP9-positive cells/field (b). **C** Representative images of T2-weighted images in each group at 12 h. Quantification of the volume of hyperintensity around the injured tissue at 12 h (b). **D** Representative images of diffusion-weighted imaging (DWI) in each group at 12 h (a). Quantification of the apparent diffusion coefficient (ADC) signal around the injured tissue at 12 h (b). (Ab), (Cb), (Db) Data are presented as the means ± SDs (n = 5 mice). ^*^P < 0.05, ^***^P < 0.001 and ^****^P < 0.0001 were determined using one-way ANOVA with Tukey’s post hoc test. (Bb) The data are presented as the means ± SDs (n = 15 fields of 3 mice). ^**^P < 0.01 and ^***^P < 0.001 were determined using one-way ANOVA with Tukey’s post hoc test

BBB permeability following TBI was estimated by the extravasation of EB dye [45–47]. Compared with those in the TBI control group, the EB leakage in the Pol, T1AM and Pol/T hydrogel-treated TBI group was inhibited (Fig. 4A). The mice in the rewarming and delay groups had greater levels of EB in the brain than did those in the Pol/T hydrogel group (Additional file 1: Figure S14A). BBB permeability was greatest in the ipsilateral hemisphere in the Pol/T hydrogel-treated TBI mice, which suggested that the Pol/T hydrogel induced timely and long-lasting local hypothermia and effectively maintained BBB integrity.

Matrix metalloproteinases (MMPs) are also associated with increased BBB permeability, which is indicative of hemorrhagic potential and the extent of brain edema [42]. MMP9 levels were investigated in the cerebral cortex (Fig. 4B and Additional file 1: Figure S14B). In the sham group, the number of MMP9-positive cells was minimal, but the number of MMP9-positive cells in the TBI group gradually increased. As shown in Fig. 4, the lowest MMP9 expression was observed in the Pol/T hydrogel group compared with the other groups. The rewarming and delayed treatment groups were used to further test the curative effect of the Pol/T hydrogel (Additional file 1: Figure S14B). Both the rewarming and delayed groups exhibited greater MMP9 expression than did the Pol/T hydrogel group. The above results demonstrated that the Pol/T hydrogel effectively induced timely and long-lasting local hypothermia and prevented brain impairment after TBI injury. To further explore the ability of the Pol/T hydrogel to effectively reduce the spread of secondary damage, brain edema was detected by T2-weighted and DWI of MRI at 12 h after percussion injury. As shown in Fig. 4C and Additional file 1: Figure S14C, brain edema centered on the percussion site was evident in the TBI control and rewarming groups. Compared with those in the TBI control and rewarming groups, the high-intensity area in the Pol hydrogel, free T1AM, delay and Pol/T hydrogel groups was restricted to a relatively small region. The contusion volume in the TBI mice was reduced in the Pol, T1AM and delay treatment groups. This phenomenon suggested that the Pol hydrogel, T1AM and delay treatments had different degrees of neuroprotection. Most importantly, the volume of edema surrounding the damaged area was the lowest in the Pol/T hydrogel group.

The dispersion of water molecules on the surface of the cerebral cortex was further evaluated by measuring the apparent diffusion coefficient (ADC) values of DWI to determine the edema of brain tissue given that the ADC values reflect the variation trend in edema with high sensitivity (Fig. 4D; Additional file 1: Figure S14D) [48]. A decrease in the apparent diffusion coefficient (ADC) indicated that cytotoxic edema gradually occurred and became more aggravated with the release of neurotoxic substances [49]. The apparent diffusion coefficient (ADC) values of the mice in the Pol hydrogel, free T1AM and delay groups were greater than those in the TBI control and rewarming groups. Consistent with the trend of hyperintensity volume in T2-weighted images, the ADC value was the highest in mice treated with the Pol/T hydrogel (Fig. 4D). We also assessed brain edema in different groups after 12 h by measuring brain water content, and these results were consistent with the T2-weighted MRI results (Additional file 1: Figure S15). Overall, the Pol/T hydrogel effectively reduced brain edema and protected BBB integrity.

### Anti-inflammatory effect of local hypothermia induced by the Pol/T hydrogel

**Fig. 5:**
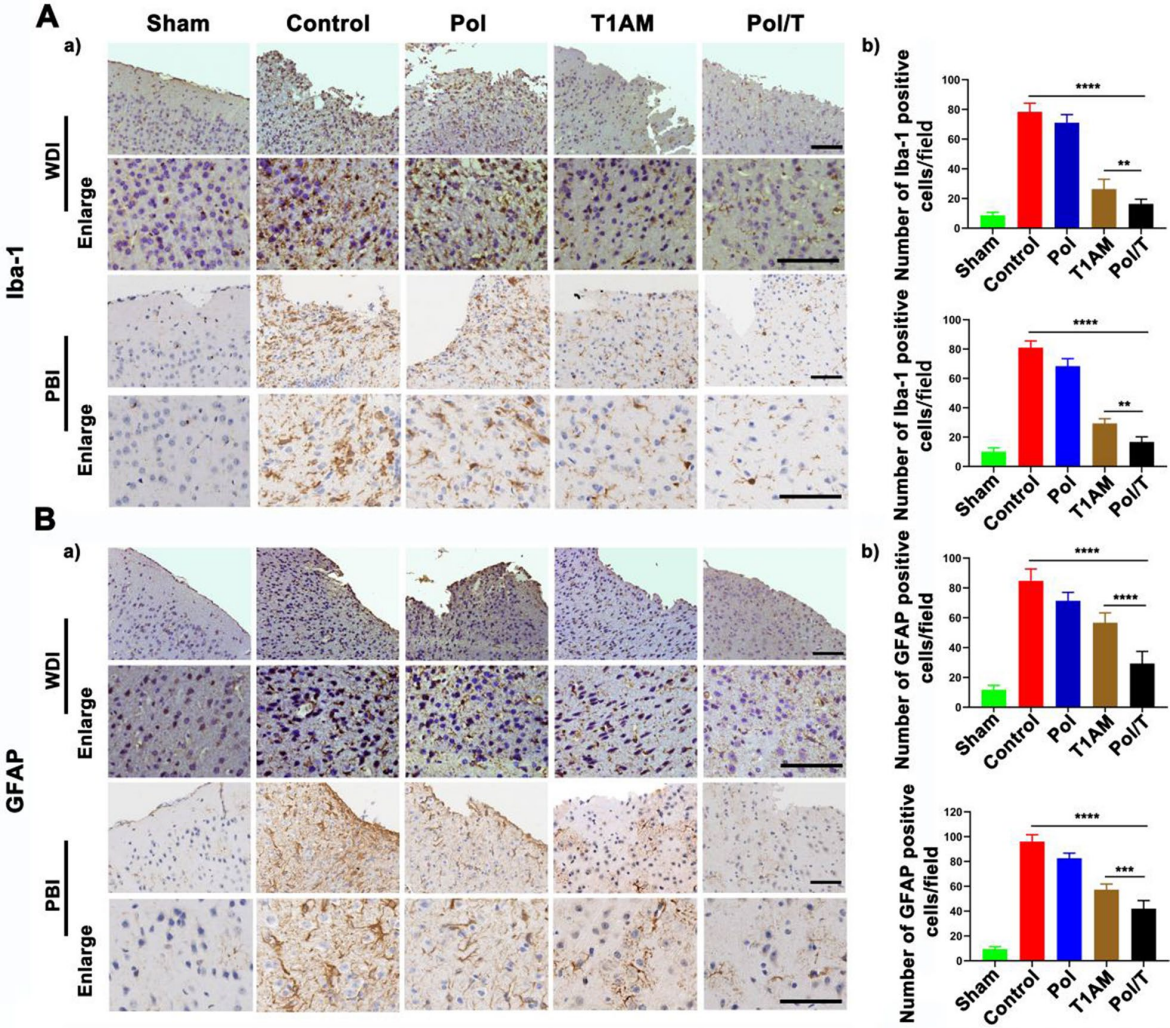
Analysis of neuroinflammation in response to different treatments. **A** Expression of ionized calcium binding adapter molecule 1 (Iba-1) in microglia in injured tissue 7 days after TBI. Scale bar = 100 μm (a). Number of Iba-1-positive cells/field (b). **B** Expression of glial fibrillary acidic protein (GFAP) in astrocytes in injured tissue at 7 d. Scale bar = 100 μm (a). Number of GFAP-positive cells/field (b). Normality was checked using the Shapiro‒Wilk test. ^**^P < 0.01, ^***^P < 0.001 and ^****^P < 0.0001 were determined by one-way ANOVA with Tukey’s post hoc test. (**C**a–**C**d) Data are presented as the means ± SDs (n = 3 mice). Normality was checked using the Shapiro‒Wilk test. ^**^P < 0.01, ^***^P < 0.001 and ^****^P < 0.0001 were determined using one-way ANOVA with Tukey’s post hoc test

The inflammatory response plays important roles in secondary damage after TBI injury [47]. Astrocytes and microglia are quickly activated, and cell bodies become enlarged and branched after brain tissue is damaged. Iba-1 is an indicator of activated microglia, and GFAP is an indicator of astrocyte activity [50]. The expression of these genes was detected by IHC at 7 d and 21 d after injury. As shown in Fig. 5A, B, we observed that Iba-1 and GFAP expression on the edge of the injured area was greater in mice in the TBI control group than in those in the sham group at 7 d postinjury, which was consistent with the results described above. Compared with those in the TBI control group, Iba-1 and GFAP expression was decreased in the Pol hydrogel, free T1AM and Pol/T hydrogel groups. As shown in Additional file 1: Figure S17A and B, compared with those in the rewarming group, Iba-1 and GFAP expression was decreased in the delay and Pol/T hydrogel groups. Importantly, both Iba-1 and GFAP expression surrounding the TBI injury site was lower in the Pol/T hydrogel group than in the other treatment groups at 7 d postinjury. Furthermore, GFAP or Iba-1 expression was lower at 21 d than at 7 d in all groups except the sham group because of the self-healing ability of the mice after TBI. More notably, the TBI mice treated with the Pol/T hydrogel had the fewest GFAP- and Iba-1-positive cells among the other treated TBI mice at 21 d postinjury (Additional file 1: Figure S17C). These results show that timely and long-lasting local hypothermia induced by the Pol/T hydrogel inhibited the chronic secondary astrogliosis inflammatory response at the TBI-injured site.

TBI induced an acute increase in proinflammatory cytokine: tumor necrosis factor-α (TNF-α), interleukin-1β (IL-1β), interleukin-6 (IL-6) and chemokine1 (CXCL1) production throughout the brain (Additional file 1: Figures S16 and S17D) [50]. According to ELISA analysis of protein levels in brain tissue 12 h posttrauma, durable and stable local hypothermia induced by the Pol/T hydrogel more significantly decreased inflammatory factor expression than transitory whole-body hypothermia via free T1AM. Taken together, the hypothermia induced by the Pol/T hydrogel effectively inhibited the inflammatory response in mice after TBI.

### Promotion of functional recovery in mice after TBI

**Fig. 6:**
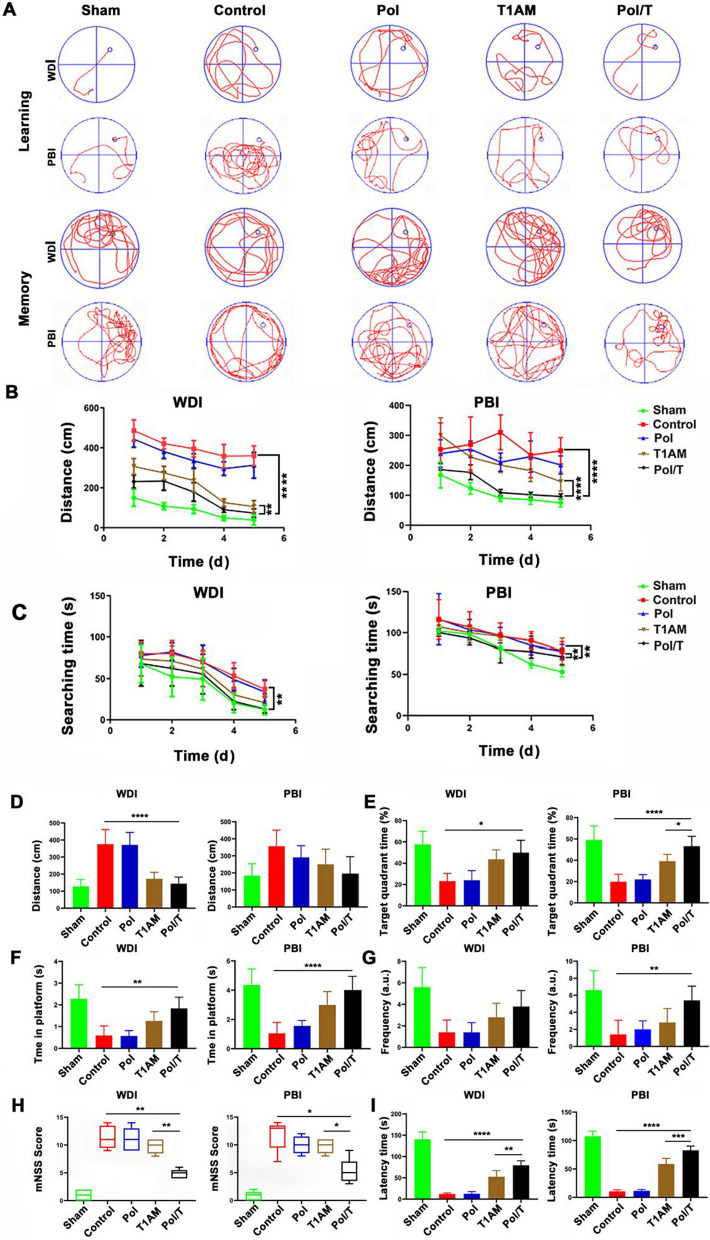
Functional recovery assessments by Morris water maze test, modified neurological severity score (mNSS) and wire hanging test on 21 d after TBI. **A** Computer printouts of the swimming trajectories during the learning phase and memory phase. **B** Swimming distance to the platform and **C** searching time for the platform distance at the last trial each day during 5 d of training. **D** Swimming distance to the platform, **E** target quadrant time, **F** duration time on the platform and **G** frequency on the platform. **H** mNSS scores. **I** Motor function was evaluated by the wire hanging test. **B**–**G**, **I** Data were presented as means ± SD (n = 5 mice). Normality was checked using Shapiro–Wilk test. ^*^P < 0.05, ^**^P < 0.01, ^***^P < 0.001 and ^****^P < 0.0001 were determined using one-way ANOVA with Tukey’s post hoc test (consistent with normal distribution) and Kruskal–Wallis test (did not consistent with normal distribution). **H** The data of mNSS were presented as the median with range (n = 5 mice). ^*^P < 0.05 and ^**^P < 0.01 were determined using Kruskal–Wallis test. Compared with T1AM and Pol/T groups

The Morris water maze test was used to assess the long-term learning and memory abilities of TBI mice (Fig. 6A–G). The Pol/T group’s mean distance was not more than 104.20 cm (WDI) or 42.63 cm (PBI) greater than that of the T1AM group [WDI: difference (95% CI): 243.10 cm, (211.20, 274.90), PBI: difference (95% CI): 129.20 cm, (80.07, 178.30)] during the first 5 d of training (Fig. 6B). The Pol/T group’s mean time was not more than 35.43 s (WDI) and 22.34 s (PBI) greater than the T1AM group's mean [WDI: difference (95% CI): 19.97 s, (4.52, 35.43), PBI: difference (95% CI): 13.11 s, (3.88, 22.34)] during the first 5 d of training (Fig. 6C). On the last day, the platform was removed, and the memory capacity of each mouse was evaluated. Among the groups that underwent actual TBI, the mice treated with the Pol/T hydrogel traveled the shortest distance to the original position of the platform (Fig. 6D). Similarly, the amount of time spent in the target quadrant, the amount of time spent in the platform area and the frequency of platform crossings were greater in the Pol/T hydrogel-treated TBI group than in the other groups subjected to actual TBI (Fig. 6E–G). The mNSS was adopted to evaluate motor and sensory function as well as reflex and balance after TBI. A high score indicated severe damage in the TBI mice [51]. Compared with those in the TBI, rewarming and Pol groups, the mNSSs were lower in the free T1AM-, delay- and Pol/T hydrogel-treated TBI group (Fig. 6H). Compared with the other groups, the Pol/T hydrogel-treated mice had the lowest mNSS, except for those in the sham group, demonstrating that the Pol/T hydrogel-treated TBI mice exhibited enhanced neurological recovery. The hanging-wire-grip test was used to assess motor strength after Pol/T hydrogel treatment. As shown in Fig. 6I, the Pol/T hydrogel-treated TBI mice exhibited greater endurance than the mice in the other treatment groups, indicating that the motor strength of the TBI mice was enhanced after Pol/T hydrogel treatment. Compared with the rewarming and delayed groups, the Pol/T hydrogel-treated TBI group exhibited the greatest functional recovery (Additional file 1: Figure S18). These results suggested that the injection of the Pol/T hydrogel into TBI mice could efficiently enhance the behavior of these animals.

## Discussion

TBI accounts for millions of deaths each year worldwide and is one of the leading causes of death and disability in young people [1]. Studies have shown that secondary brain injury is a significant risk factor leading to an acute inflammatory response, which induces breakdown of the BBB, edema formation and swelling, local electrolyte imbalance, and persistent neurological impairment. As the primary injury is irreversible, preventing secondary injury has significance in clinical practice [4, 5, 52]. Therapeutic hypothermia can reduce intracranial hypertension and is regarded as a neuroprotective agent to prevent secondary brain injury in TBI patients [10, 12].

Whole-body cooling and local hypothermia (including epidural placement of a cooling catheter [21], local cold fluid infusion [19] and local ice compress [22]) represent the main current therapies for hypothermia for clinical application. However, whole-body cooling has many negative effects [50, 53], and local hypothermia induced by sublow-temperature therapeutic instruments has technical barriers; thus, timely and effective local hypothermia cannot be achieved. Therefore, methods to achieve timely and long-lasting local hypothermia are urgently needed.

T1AM (a cooling agent) can bind to multiple targets to induce profound hypothermia within minutes, which is a very complicated physiological process that involves the joint action of multiple mechanisms [31, 32, 34]. Many studies have demonstrated that T1AM can stimulate cAMP production in TAAR1-expressing cells, which leads to severe hypothermia [31]. In addition to TAAR1, some studies have reported that T1AM can bind to the thermosensitive transient receptor potential melastatin 8 (TRPM8) to generate hypothermic effects [54, 55]. Neurons and astrocytes in the brain both express TAAR1 [35]. Therefore, we speculated that T1AM would be accomplished by stimulating TAAR1 to achieve hypothermia in the brain. To further clarify whether TAAR1 is indispensable for the hypothermic effect, we used the TAAR1 antagonist *N*-(3-ethoxy-phenyl)-4-pyrrolidin-1-yl-3-trifluoromethyl-benzamide (EPPTB) to block TAAR1 in TBI mice. Compared with those in the T1AM group, the brain temperature decreased less in the EPPTB + free T1AM group, suggesting that hypothermia was a cerebral TAAR1-mediated effect (Additional file 1: Figure S19). Moreover, the above results also indicated that multiple pathways might be involved in the development of hypothermia in the brain induced by T1AM. In addition, the rapid metabolism of T1AM [29, 32] limits its concentration in the brain. T1AM targets are widely distributed in various organs, and the negative heart effect is especially significant given that a high dose of T1AM induces death in animal models. In this study, we used T1AM to induce profound hypothermia and hydrogels embedded with T1AM (Pol/T hydrogel) to change the route of administration of T1AM to produce hypothermia in the brain and reduce side effects. The Pol/T hydrogel has several unique features that induce timely, effective, and persistent local hypothermia while avoiding systemic side effects. Our results showed that the Pol/T hydrogel could effectively protect BBB integrity, prevent cell death, reduce the inflammatory response and brain edema, and promote functional recovery in mice after TBI. The drug release characteristics of the Pol/T hydrogel are important for persistent local hypothermia. To create a similar human TBI environment, we collected CSF from clinical TBI patients to test the release of T1AM from the Pol/T hydrogel. Approximately 85% of the T1AM was released from the Pol/T hydrogel during incubation in CSF within 12 h (Fig. 1F). The amount of T1AM released from the Pol/T hydrogel was tested 12 h after the injection of the Pol/T hydrogel into the injured cerebral hemisphere. T1AM released from the Pol/T hydrogel could not reach the hypothalamus for 12 h (Additional file 1: Figure S4). These results suggested that local application of the Pol/T hydrogel could induce hypothermia for up to 12 h and did not cause systemic cooling of the hypothalamus. The brain temperature of the TBI male mice treated with the Pol/T hydrogel decreased to hypothermia, while the body temperature was within the normal range (Fig. 2B and C). The brain and body temperatures of male and female mice treated with the Pol/T hydrogel did not differ (Additional file 1: Figure S6). We measured changes in brain temperature at 1 h intervals in the sham group of mice treated with Pol and Pol/T. The results showed that the brain temperature of the sham group treated with the Pol/T hydrogel exhibited hypothermia for 12 h, consistent with that of the Pol/T hydrogel-treated TBI group, suggesting that some specific receptors of T1AM were distributed in the brain (Additional file 1: Figure S7). At the same time, IHC staining revealed no difference in TUNEL, Bcl-2, Bax or MMP9-positive brain tissue between the sham controls treated with Pol or Pol/T and the sham controls (Fig. 3), suggesting that there were no negative effects on brain tissue. Due to the systemic negative effects of whole-body hypothermia and the low operability of local hypothermia, hypothermia is greatly limited in the clinic [56]. The advantages of hydrogels are that they can be used as drug carriers to achieve timely, effective and long-lasting local hypothermia. This new method can avoid multiple problems associated with existing methods. We used the Pol/T hydrogel to treat TBI mice and closely monitored their vital signs. We observed that the brain temperature markedly decreased to a low level, but the body temperature was maintained at a normal level in the mice. The cooling time was longer than that of traditional pharmacologically induced hypothermia, and the rewarming process was gradual without dramatic changes. As shown in Fig. 2D–G, heart rate, systolic aortic pressure, respiratory rate, oxygen saturation, and the number of platelets and red blood cells did not differ between the Pol/T hydrogen-treated group and the sham group. However, compared with those in the sham group, heart rate, systolic aortic pressure, respiratory rate, and oxygen saturation were significantly lower in the T1AM group, which was consistent with previous reports [31]. Importantly, the vital signs and behavioral profiles of the TBI mice that received the Pol/T hydrogel remained more stable than those of the mice treated with T1AM throughout the process. These results demonstrated that the Pol/T hydrogel induced prolonged and effective selective brain hypothermia and maintained stability throughout the whole body by preventing systemic negative effects around the cooling time. We found that this local hypothermia had a great protective effect on neurons from damage induced by TBI. Neuroapoptosis is an inevitable process and the main form of neurodeath in the acute phase of secondary brain injury when the cerebral cortex is affected. In the two TBI mouse models, TUNEL-positive cells and the Bcl-2/Bax ratio were examined to assess neuronal apoptosis. Compared with those in the TBI control and rewarming groups, the Pol/T hydrogel induced local hypothermia and effectively protected neurons from damage caused by TBI (Fig. 3). Moreover, we found that delayed hypothermia (6 h after trauma injection of the Pol/T hydrogel) failed to mitigate neuronal damage. These results showed that the time window was the key to hypothermia therapy. In addition, the earlier therapy started, the better the effect was. The timely local hypothermia induced by this hydrogel is perfectly consistent with this treatment concept.

Destruction of the BBB is the essential basis of brain edema after TBI [51]. Hypothermia can protect the BBB and is associated with a concomitant decrease in BBB permeability, structural changes in capillary blood vessels, and upregulation of MMP9 activity [13]. We showed that the local hypothermia induced by the Pol/T hydrogel enhanced the integrity of the BBB and decreased brain edema. In our study, MMP9 was upregulated after TBI, and this increase was significantly inhibited by Pol/T hydrogel-induced local hypothermia. Pol/T hydrogel treatment inhibited EB leakage and restricted the leakage area to the primary lesion site, suggesting that the Pol/T hydrogel induced local hypothermia and effectively maintained BBB integrity. T2-weighted and DWI results suggested that the Pol/T hydrogel significantly reduced the level of brain edema after TBI. Compared with those in the Pol/T hydrogel group, the groups that were rewarmed after Pol/T hydrogel administration or delayed Pol/T hydrogel treatment exhibited some degree of edema and impaired BBB integrity. However, Pol/T hydrogel-induced local hypothermia was more effective due to the timely and long-lasting hypothermia and the milder rewarming process.

The inflammatory response plays an important role in secondary damage after TBI injury [50]. Hypothermia decreases the inflammatory production of different proinflammatory factors and the activation of microglia and astrocytes [41]. Astrocytes and microglia are quickly activated, and the cell bodies become enlarged and branched after brain tissue damage. Iba-1 is an indicator of GFAP activity in astrocytes. Our studies showed that the local hypothermia induced by the Pol/T hydrogel attenuated the expression of proinflammatory factors and restrained the activation of microglia and astrocytes.

Clinical studies have shown that neurologic function deficits are the most severe sequelae caused by TBI [1, 3]. It is essential to promote the recovery of neurologic function and enhance quality of life. In animal studies, hypothermia therapy has been shown to have a great protective effect on sensorimotor function in hypoxia-ischemic mice. The local hypothermia induced by the Pol/T hydrogel effectively restored the long-term learning and memory abilities of TBI mice. The sensitivity of the reaction and muscular strength were also improved by the Pol/T hydrogel. These results suggest that long-term functional benefits could be achieved after local hypothermia was induced by the Pol/T hydrogel.

In summary, injectable and biodegradable refrigerated Pol/T hydrogels were developed to induce local hypothermia for TBI mouse treatment. Sol Pol/T was directly injected into the surgical cavity after TBI to form the Pol/T hydrogel, which was biodegraded to release T1AM to achieve local hypothermia in the brain. The temperature of the brain was maintained under hypothermia for 12 h, and the body temperature did not change after treatment with the Pol/T hydrogels. Moreover, no severe side effects were observed after treatment with the Pol/T hydrogels. More importantly, the Pol/T hydrogels effectively protected BBB integrity, prevented cell death, reduced the inflammatory response and brain edema, and promoted functional recovery after TBI in mice. However, there are still many problems to be solved prior to clinical application. The optimal duration of Pol/T hydrogel administration and the duration of local hypothermia required to achieve the best therapeutic effect on TBI require further investigation. Overall, these findings demonstrate that the Pol/T hydrogel provides a potential local hypothermia approach for preventing secondary injury after TBI.

### Supplementary Information

**Additional file 1: Figure S1.** The heating coil installation and temperature measurement. **Figure S2.** Average levels of T1AM in cerebral hemisphere measured by HPLC‐MS/MS. **Figure S3.** The security of the Pol hydrogel by measuring cell viability. **Figure S4.** Average levels of T1AM in hypothalamus measured by HPLC‐MS/MS. **Figure S5.** Survival curve for the mice treated with different dose of T1AM, intraperitoneal injection. (n = 5 mice). **Figure S6.** Body and brain temperature of the male and female mice treated with the Pol/T hydrogel in WDI models. **Figure S7.** The temperature variation in the brain of mice in sham model received the Pol/T and Pol treatments along with the time. **Figure S8.** The hemoglobin content of brain after 12 h post-TBI in different groups. **Figure S9.** (A) Expression of Tunel, Bcl-2, Bax, MMP9 in brain tissue at 21 d. Scale bar = 100 μm. (B) Number of Tunel + cell death/field (a), Bcl-2 positive cells/field (b), Bax positive cells/field (c) and MMP9 positive cells/field (d). (B) Data were presented as means ± SDs (5 technical replicates averaged for each 3 mice/group). Normality and homogeneity of variance was checked using Shapiro–Wilk test. Statistical significance of the results was determined using one-way ANOVA with Tukey’s post hoc test. **Figure S10.** Brain section from TBI mice, taken at day 21 post-injury, with indicated treatments administered at 21 post-injury time point. **Figure S11.** (A) Representative fields of cells positive for TdT-mediated dUTP nick-end labeling (Tunel) stain in the different groups. Scale bar = 50 μm. (B) Quantified bar graph of Tunel + cell death. ****P < 0.0001 were performed using one-way ANOVA with Tukey’s post hoc test. **Figure S12.** (A) The ratio of Bcl-2/Bax-positive cells/field in WDI models. (B) The ratio of Bcl-2/Bax-positive cells/field in PBI models. Data were represented as means ± SDs (n = 15 fields of 3 mice). ^*^P < 0.05, ^**^P < 0.01 and ^****^P < 0.0001 were performed using Kruskal–Wallis test. **Figure S13.** (A) Representative fields of cells positive for Tunel stain in the different groups. Scale bar = 50 μm. (B) Quantified bar graph of Tunel + cell death. (C) Representative fields of Bcl-2 staining around the injury site in different groups (a) Representative fields of Bax staining around the injury site in different groups (b). Scale bar = 100 μm. (D) The ratio of Bcl-2/Bax-positive cells/field. (B) Data are represented as means ± SDs (5 technical replicates averaged for each 3 mice/group). ^**^P < 0.01 and ^****^P < 0.0001 were performed using one-way ANOVA with Tukey’s post hoc test. (D) Data are represented as means ± SDs (5 technical replicates averaged for each 3 mice/group). ^**^P < 0.01, ^***^P < 0.001 and ^****^P < 0.0001 were performed using Kruskal–Wallis test. **Figure S14.** Local hypothermia induced by the Pol/T hydrogel protected the integrity of the BBB and educed brain edema in the TBI mouse model. **Figure S15.** Brain water content. Data were presented as means ± SDs (n = 5 mice). Normality was checked using Shapiro–Wilk test. ^*^P < 0.05, ^**^P < 0.01 and ^****^P < 0.0001 were performed using one-way ANOVA with Tukey’s post hoc test. **Figure S16.** Levels of tumor necrosis factor-α (TNF-α), interleukin-1β (IL-1β), interleukin-6 (IL-6) and chemokine1 (CXCL1) in injured tissue by enzyme linked immunosorbent assay (ELISA) at 12 h after TBI. Data were presented as means ± SD (n = 3 mice). **Figure S17.** Analysis of neuroinflammation in response to different treatments. **Figure S18.** Functional recovery assessments by Morris water maze test, mNSS score and wire hanging test on 21 d after TBI. **Figure S19.** Brain temperature variation of Sham group, T1AM group and T1AM + EPPTB group. Data were presented as means ± SDs (n = 4 mice).

**Additional file 2: Movie S1.** The behavior of TBI models treated with T1AM, intraperitoneal injection.

**Additional file 3: Movie S2.** The behavior of TBI models treated with Pol/T hydrogel.

## Data Availability

All the other data supporting the findings of this study are available within the article and its Supplementary Information Files, or from the corresponding authors upon reasonable request.
