# Supplementary material for: An injectable refrigerated hydrogel for inducing local hypothermia and neuroprotection against traumatic brain injury in mice
Source: J Nanobiotechnology. 2024 May 15;22:251. doi: 10.1186/s12951-024-02454-z (PMC11095020; doi:10.1186/s12951-024-02454-z)
Supplement: Supplementary file 1 — Additional file 1: Figure S1. The heating coil installation and temperature measurement. Figure S2. Average levels of T1AM in cerebral hemisphere measured by HPLC‐MS/MS. Figure S3. The security of the Pol hydrogel by measuring cell viability. Figure S4. Average levels of T1AM in hypothalamus measured by HPLC‐MS/MS. Figure S5. Survival curve for the mice treated with different dose of T1AM, intraperitoneal injection. (n = 5 mice). Figure S6. Body and brain temperature of the male and female mice treated with the Pol/T hydrogel in WDI models. Figure S7. The temperature variation in the brain of mice in sham model received the Pol/T and Pol treatments along with the time. Figure S8. The hemoglobin content of brain after 12 h post-TBI in different groups. Figure S9. (A) Expression of Tunel, Bcl-2, Bax, MMP9 in brain tissue at 21 d. Scale bar = 100 μm. (B) Number of Tunel + cell death/field (a), Bcl-2 positive cells/field (b), Bax positive cells/field (c) and MMP9 positive cells/field (d). (B) Data were presented as means ± SDs (5 technical replicates averaged for each 3 mice/group). Normality and homogeneity of variance was checked using Shapiro–Wilk test. Statistical significance of the results was determined using one-way ANOVA with Tukey’s post hoc test. Figure S10. Brain section from TBI mice, taken at day 21 post-injury, with indicated treatments administered at 21 post-injury time point. Figure S11. (A) Representative fields of cells positive for TdT-mediated dUTP nick-end labeling (Tunel) stain in the different groups. Scale bar = 50 μm. (B) Quantified bar graph of Tunel + cell death. ****P < 0.0001 were performed using one-way ANOVA with Tukey’s post hoc test. Figure S12. (A) The ratio of Bcl-2/Bax-positive cells/field in WDI models. (B) The ratio of Bcl-2/Bax-positive cells/field in PBI models. Data were represented as means ± SDs (n = 15 fields of 3 mice). *P < 0.05, **P < 0.01 and ****P < 0.0001 were performed using Kruskal–Wallis test. Figure S13. (A) Repres [file 12951_2024_2454_MOESM1_ESM.docx]

**An injectable refrigerated hydrogel for inducing local hypothermia and neuroprotection against traumatic brain injury in mice**

Yuhan Han^1,3†^, Zhengzhong Han^4†^, Xuyang Huang^2,5†^, Shanshan Li^6^, Guoliang Jin^7^, Junfeng Feng^3*^, Decheng Wu^1*^, and Hongmei Liu^1*^

^*^Correspondence: liuhm@sustech.edu.cn; wudc@sustech.edu.cn; fengjfmail@163.com

^†^Yuhan Han, Zhengzhong Han and Xuyang Huang contributed equally to this work

^1^Department of Biomedical Engineering, Southern University of Science and Technology, Shenzhen, Guangdong, 51800, China

^2^Institute of Nervous System Diseases, Xuzhou Medical University, Xuzhou, Jiangsu, 221000, China

^3^Brain Injury Center, Ren Ji Hospital, Shanghai Jiao Tong University School of Medicine, Shanghai Institute of Head Trauma, Shanghai, 200127, China

^4^Department of Neurosurgery, Xuzhou Children's Hospital, Xuzhou, Jiangsu, 221000, China

^5^Department of Intensive Care Medicine, The Second Hospital of Jiaxing, Jiaxing, Zhejiang, 314000, China

^6^Department of Forensic Medicine, Xuzhou Medical University, Xuzhou, Jiangsu, 221000, China

^7^Department of Neurology, Affiliated Hospital of Xuzhou Medical University, Xuzhou, Jiangsu, 221000, China

**Additional information**

*
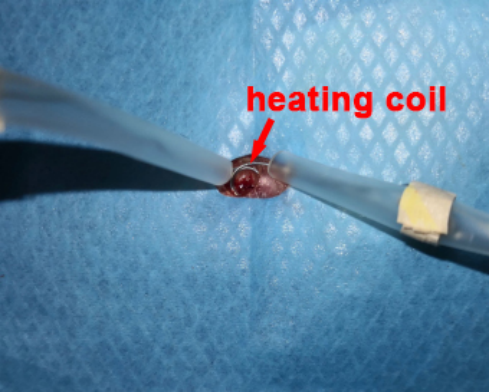
*

**Fig. S1** The heating coil installation and temperature measurement. The brain of rewarming group was continuously rewarmed by the heating coil and the temperature was measured by the coil which also has the function of measuring temperature.


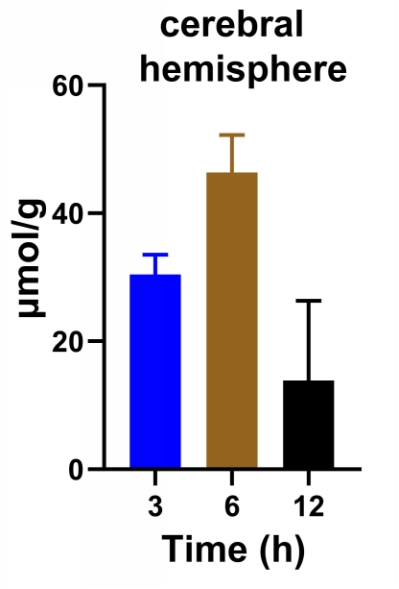


**Fig. S2** Average levels of T1AM in cerebral hemisphere measured by HPLC‐MS/MS. Data are represented as means ± SDs (n = 3 mice/time).


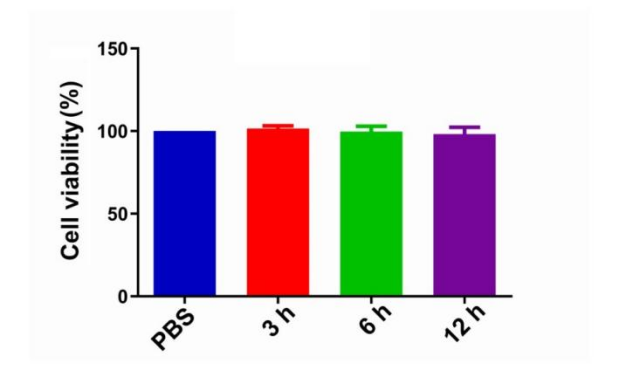


**Fig. S3** The security of the Pol hydrogel by measuring cell viability. Data are represented as means ± SDs (n = 3 ostiole/time).


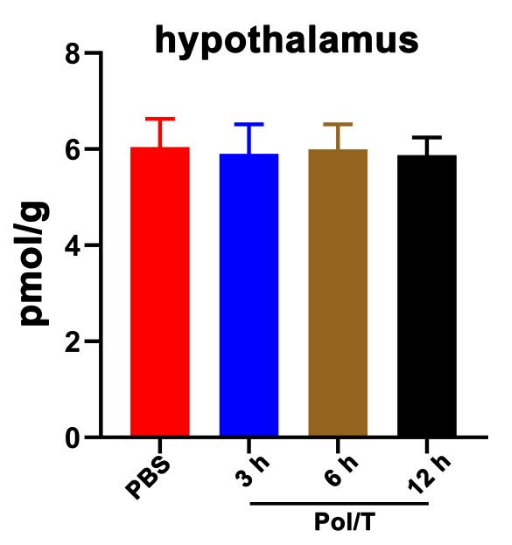


**Fig. S4** Average levels of T1AM in hypothalamus measured by HPLC‐MS/MS. Data were represented as means ± SDs (n = 3 mice).


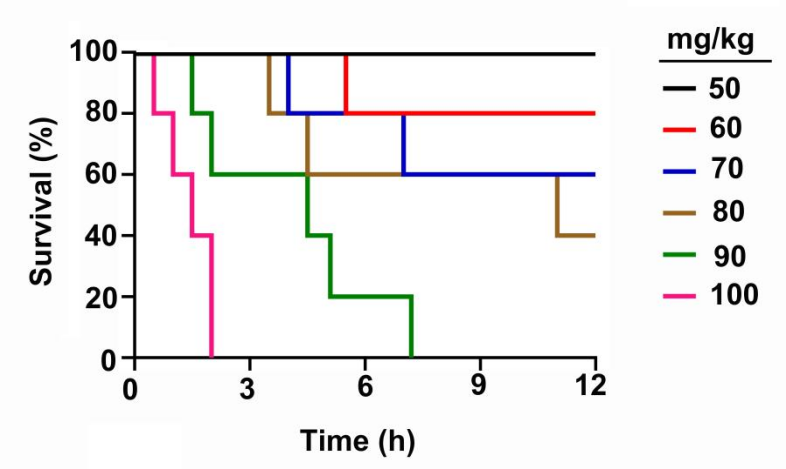


**Fig. S5** Survival curve for the mice treated with different dose of T1AM, intraperitoneal injection. (n = 5 mice).


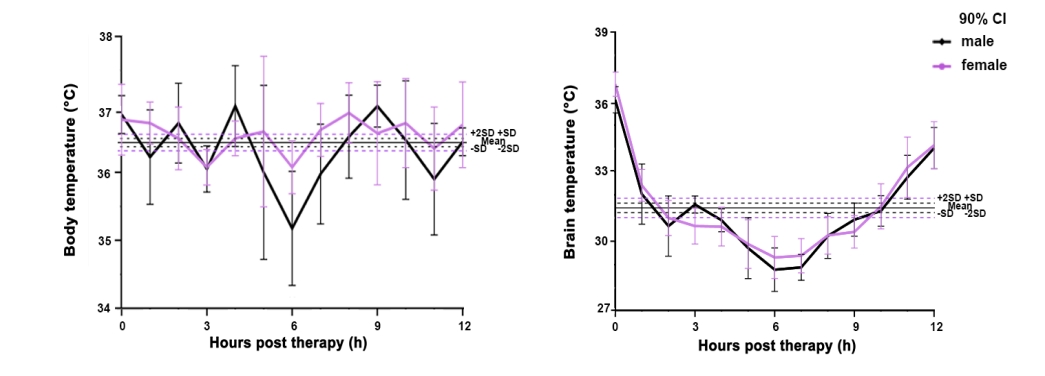


**Fig. S6** Body and brain temperature of the male and female mice treated with the Pol/T hydrogel in WDI models. Data were represented as means ± SDs (n = 4 mice). Statistical significance was performed using repeated measures of one-way ANOVA with post hoc Bonferroni analysis.


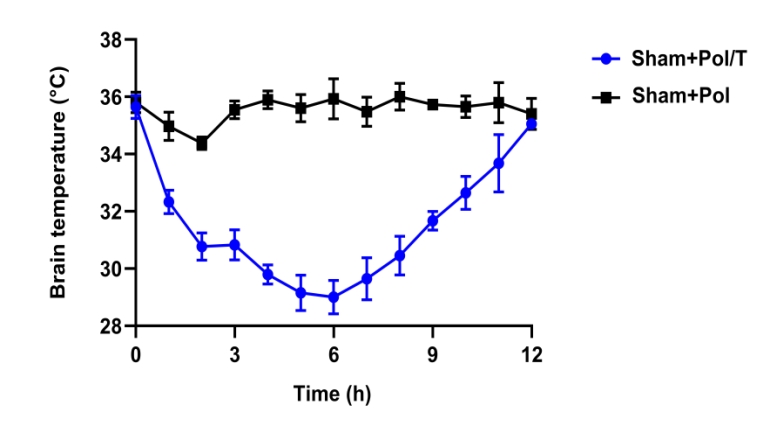


**Fig. S7** The temperature variation in the brain of mice in sham model received the Pol/T and Pol treatments along with the time. Data were represented as means ± SDs (n = 4 mice).

*
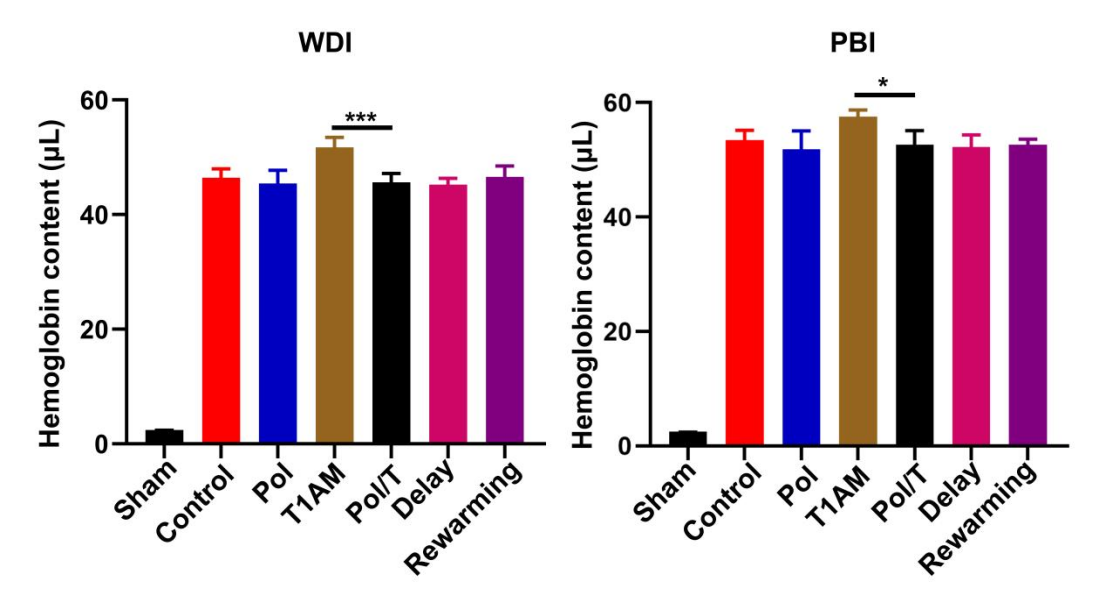
*

**Fig. S8** The hemoglobin content of brain after 12 h post-TBI in different groups. Data were represented as means ± SDs (n = 4 mice). Normality and homogeneity of variance was checked using Shapiro-Wilk test. ^*^*P* < 0.05 and ^***^*P* < 0.001 were determined using one-way ANOVA with Tukey’s post hoc test.


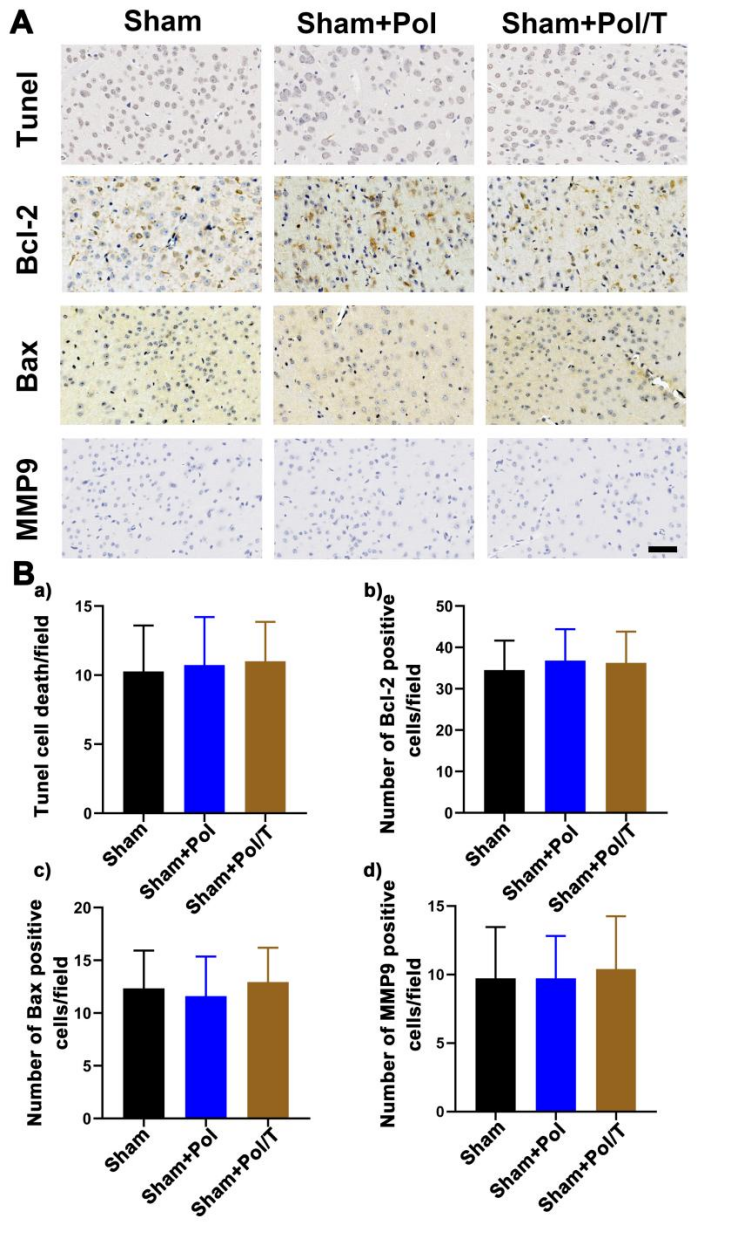


**Fig. S9** (A) Expression of Tunel, Bcl-2, Bax, MMP9 in brain tissue at 21 d. Scale bar = 100 μm. (B) Number of Tunel+ cell death/field (a), Bcl-2 positive cells/field (b), Bax positive cells/field (c) and MMP9 positive cells/field (d). (B) Data were presented as means ± SDs (5 technical replicates averaged for each 3 mice/group). Normality and homogeneity of variance was checked using Shapiro-Wilk test. Statistical significance of the results was determined using one-way ANOVA with Tukey’s post hoc test.


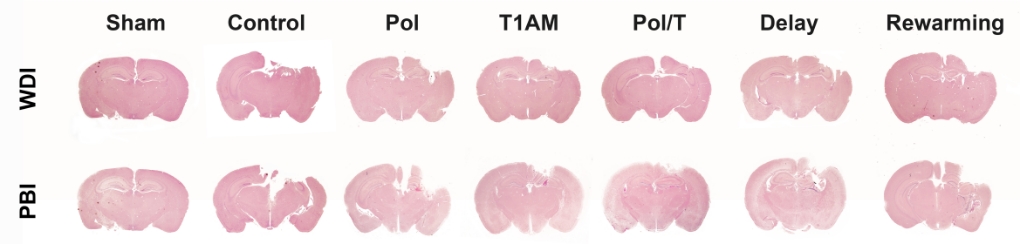


**Fig. S10** Brain section from TBI mice, taken at day 21 post-injury, with indicated treatments administered at 21 post-injury time point.


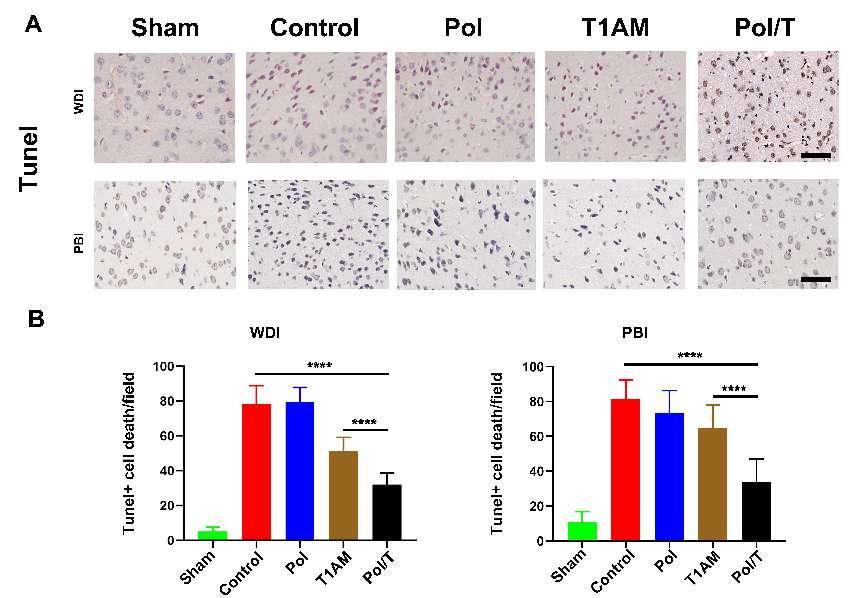


**Fig. S11** (A) Representative fields of cells positive for TdT-mediated dUTP nick-end labeling (Tunel) stain in the different groups. Scale bar = 50 μm. (B) Quantified bar graph of Tunel+ cell death. *****P*< 0.0001 were performed using one-way ANOVA with Tukey’s post hoc test.


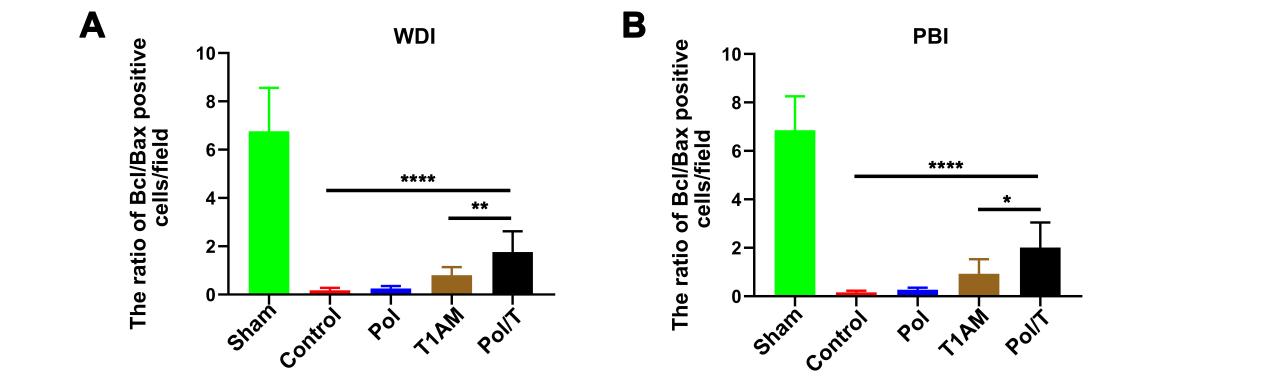


**Fig. S12** (A) The ratio of Bcl-2/Bax-positive cells/field in WDI models. (B) The ratio of Bcl-2/Bax-positive cells/field in PBI models. Data were represented as means ± SDs (n = 15 fields of 3 mice). *^*^P* < 0.05, ^**^*P* <0.01 and ^****^*P*< 0.0001 were performed using Kruskal-Wallis test.


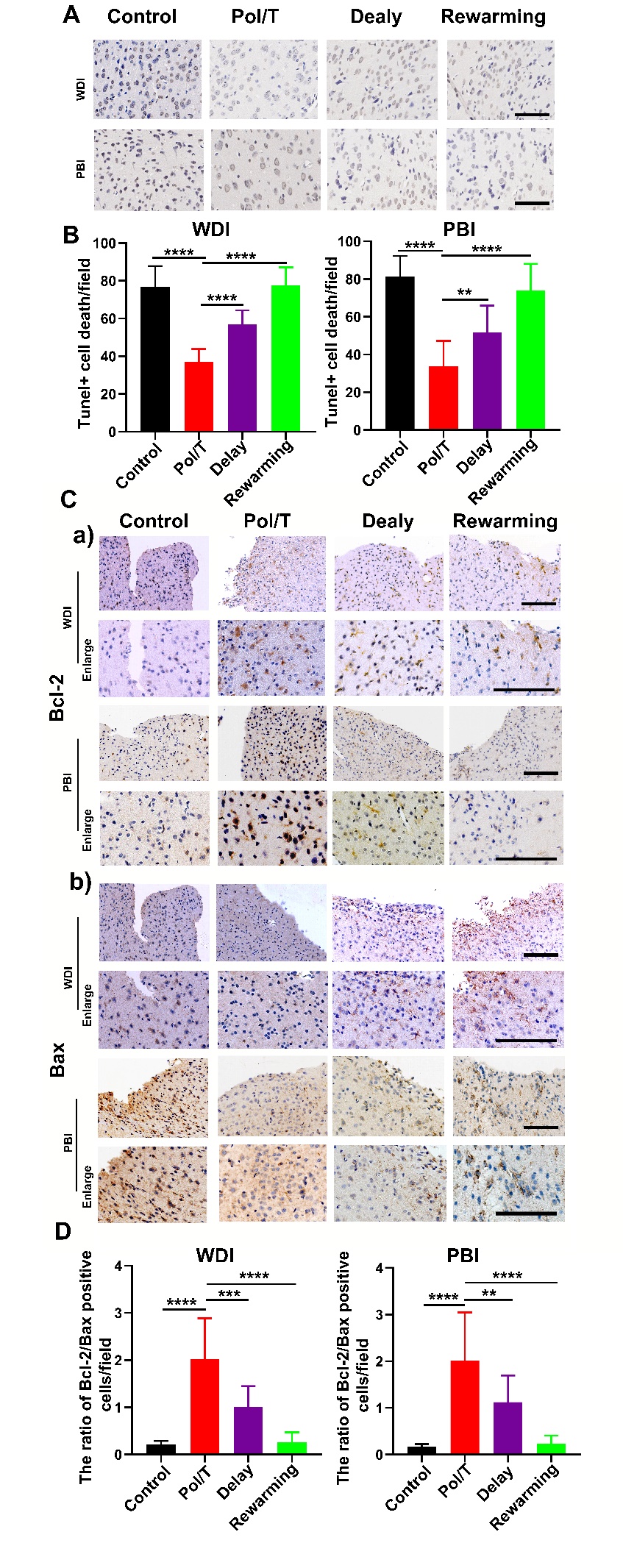


**Fig. S13** (A) Representative fields of cells positive for Tunel stain in the different groups. Scale bar = 50 μm. (B) Quantified bar graph of Tunel+ cell death. (C) Representative fields of Bcl-2 staining around the injury site in different groups (a) Representative fields of Bax staining around the injury site in different groups (b). Scale bar = 100 μm. (D) The ratio of Bcl-2/Bax-positive cells/field. (B) Data are represented as means ± SDs (5 technical replicates averaged for each 3 mice/group). *^**^P* < 0.01 and *^****^P* < 0.0001 were performed using one-way ANOVA with Tukey’s post hoc test. (D) Data are represented as means ± SDs (5 technical replicates averaged for each 3 mice/group). ^**^*P* < 0.01, ^***^*P* < 0.001 and ^****^*P* < 0.0001 were performed using Kruskal-Wallis test.


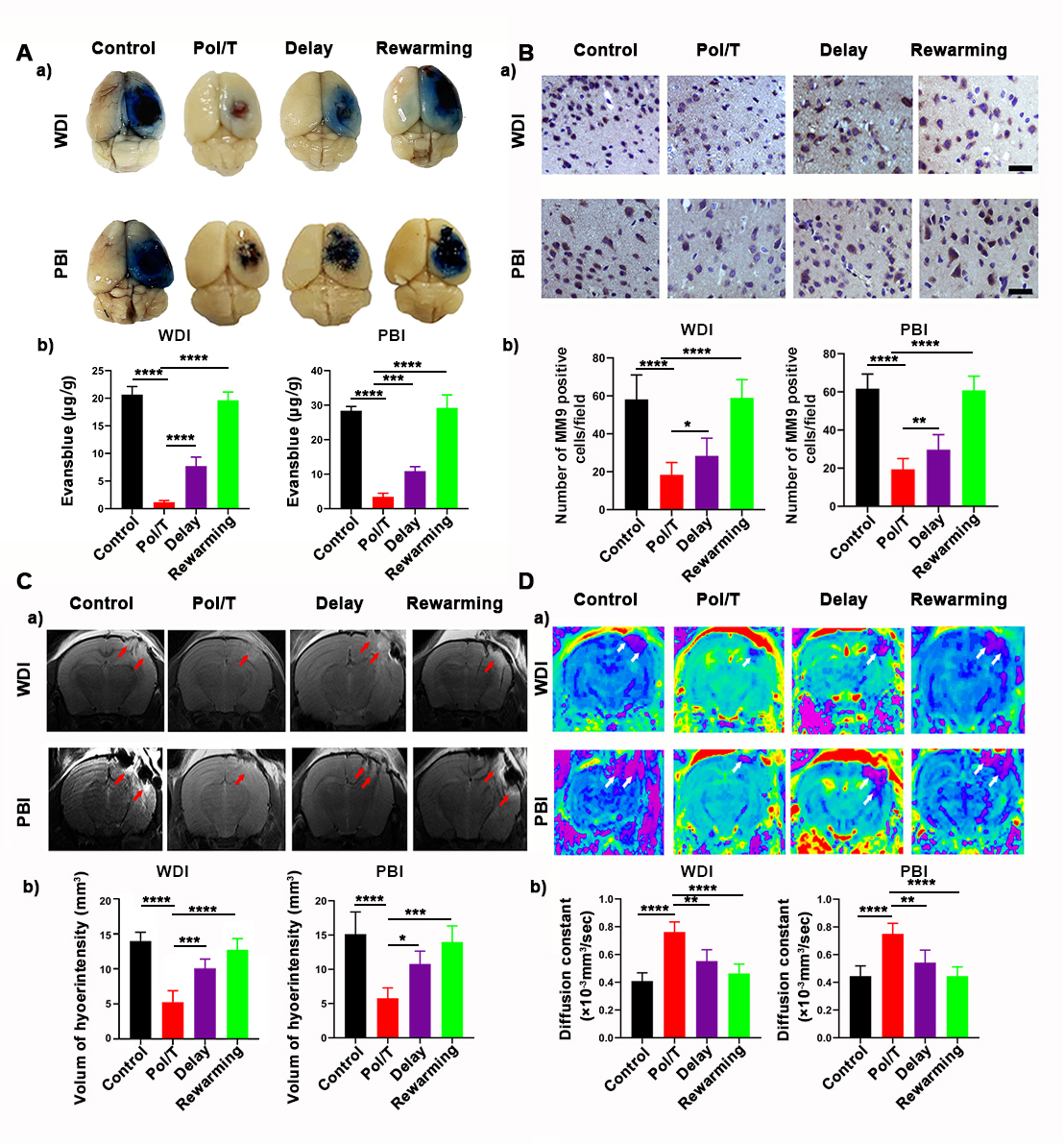


**Fig. S14** Local hypothermia induced by the Pol/T hydrogel protected the integrity of the BBB and educed brain edema in the TBI mouse model. (A) Images of EB leakage from brain capillary vessels in the right brains of mice in each group at 12 h (a). Quantification of EB leakage in each group at 12 h (b). (B) Expression of MMP9 in the injured tissue at 12 h. Scale bar = 50 μm (a). Number of MMP9-positive cells/field (b). (C) Representative images of T2-weighted in each group at 12 h. Quantification of the volume of hyperintensity around the injured tissue at 12 h (b). (D) Representative images of DWI in each group at 12 h (a). Quantification of the ADC signal around the injured tissue 12 h (b). (Ab), (Cb), (Db) Data were presented as means ± SD (n = 5 mice). Normality was checked using Shapiro-Wilk test. *^*^P* < 0.05, *^**^P* < 0.01, *^***^P* < 0.001 and *^****^P* < 0.0001 were performed using one-way ANOVA with Tukey’s post hoc test. (Bb) Data were presented as means ± SDs (5 technical replicates averaged for each 3 mice/group). Normality was checked using Shapiro-Wilk test. *^*^P* < 0.05, *^**^P* < 0.01 and *^****^P* < 0.0001 were performed using one-way ANOVA with Tukey’s post hoc test.


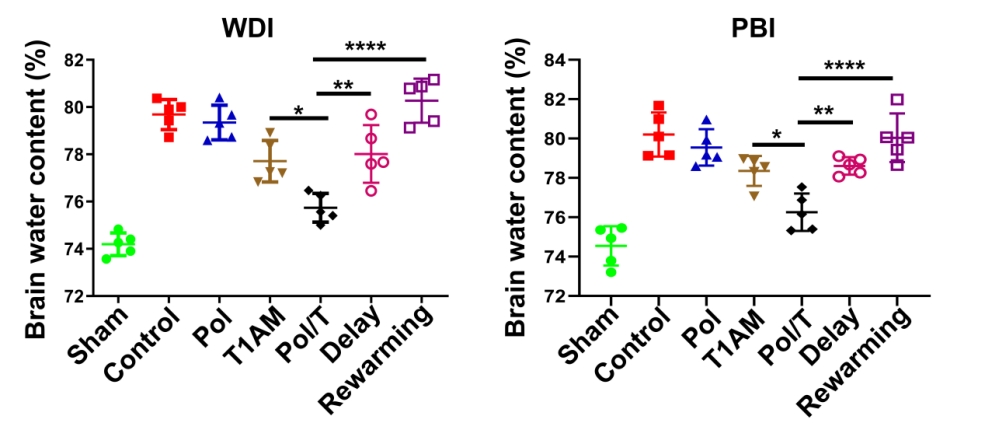


**Fig. S15** Brain water content. Data were presented as means ± SDs (n = 5 mice). Normality was checked using Shapiro-Wilk test. *^*^P* < 0.05, ^**^*P* < 0.01 and *^****^P* < 0.0001 were performed using one-way ANOVA with Tukey’s post hoc test.


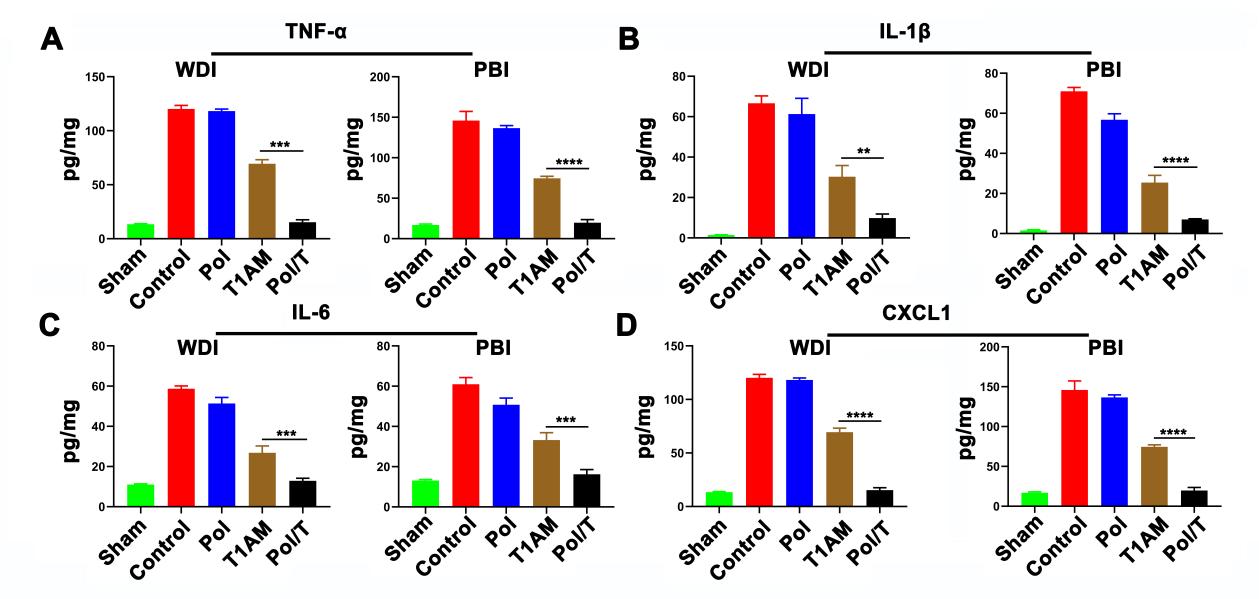


**Fig. S16** Levels of tumor necrosis factor-α (TNF-α), interleukin-1β (IL-1β), interleukin-6 (IL-6) and chemokine1 (CXCL1) in injured tissue by enzyme linked immunosorbent assay (ELISA) at 12 h after TBI. Data were presented as means ± SD (n = 3 mice). Normality was checked using Shapiro-Wilk test. ^**^*P* < 0.01, ^***^*P* < 0.001 and *^****^P* < 0.0001 were determined using one-way ANOVA with Tukey’s post hoc test.


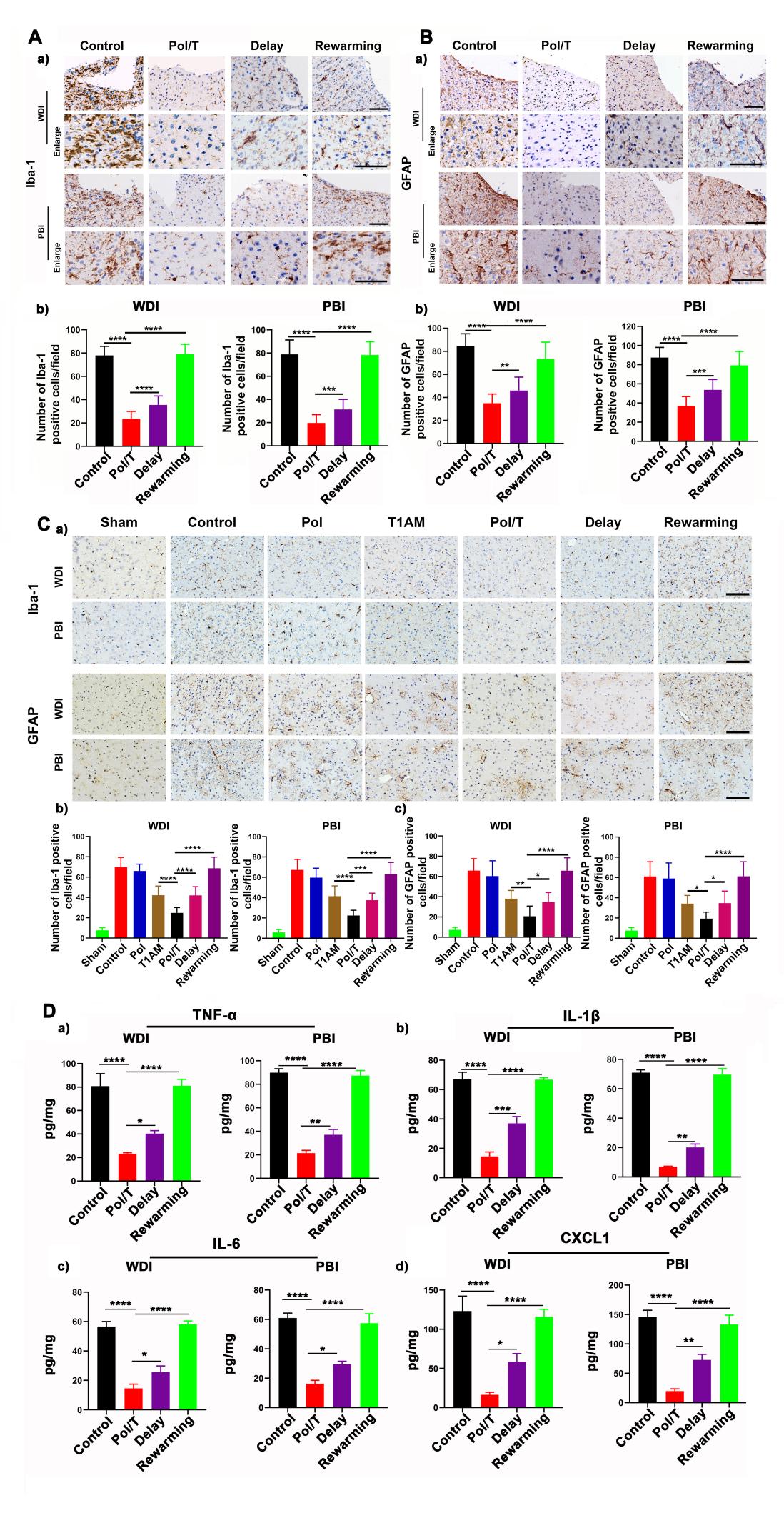


**Fig. S17** Analysis of neuroinflammation in response to different treatments. (A) Expression of Iba-1 in microglial cells in injured tissue on 7 d after TBI. Scale bar = 100 μm and Enlarge Scale bar = 50 μm (a). Number of Iba-1-positive cells/field (b). (B) Expression of GFAP in astrocytes in injured tissue at 7 d. Scale bar = 100 μm (a). Number of GFAP-positive cells/field (b). (C) Expression of Iba-1 in microglial cells and expression of GFAP in astrocytes in injured tissue on 21 d after TBI. Scale bar = 100 μm (a). Number of GFAP-positive cells/field (b). Number of Iba-1-positive cells/field (c). (D) Levels of TNF-α (a), IL-1β (b), IL-6 (c) and CXCL1 (d) in injured tissue by ELISA at 12 h after TBI. (Ab), (Bb), (Cb), (Cc) Data were presented as means ± SDs (5 technical replicates averaged for each 3 mice/group). Normality was checked using Shapiro-Wilk test. *^*^P* < 0.05, *^**^P* < 0.01, *^***^P* < 0.001and *^****^P* < 0.0001 were performed using one-way ANOVA with Tukey’s post hoc test. (Da-Dd) Data were presented as means ± SDs (n = 3 mice). Normality was checked using Shapiro-Wilk test. *^*^P* < 0.05, *^**^P* < 0.01, *^***^P* < 0.001 and ^****^*P* < 0.0001 were performed using one-way ANOVA with Tukey’s post hoc test.


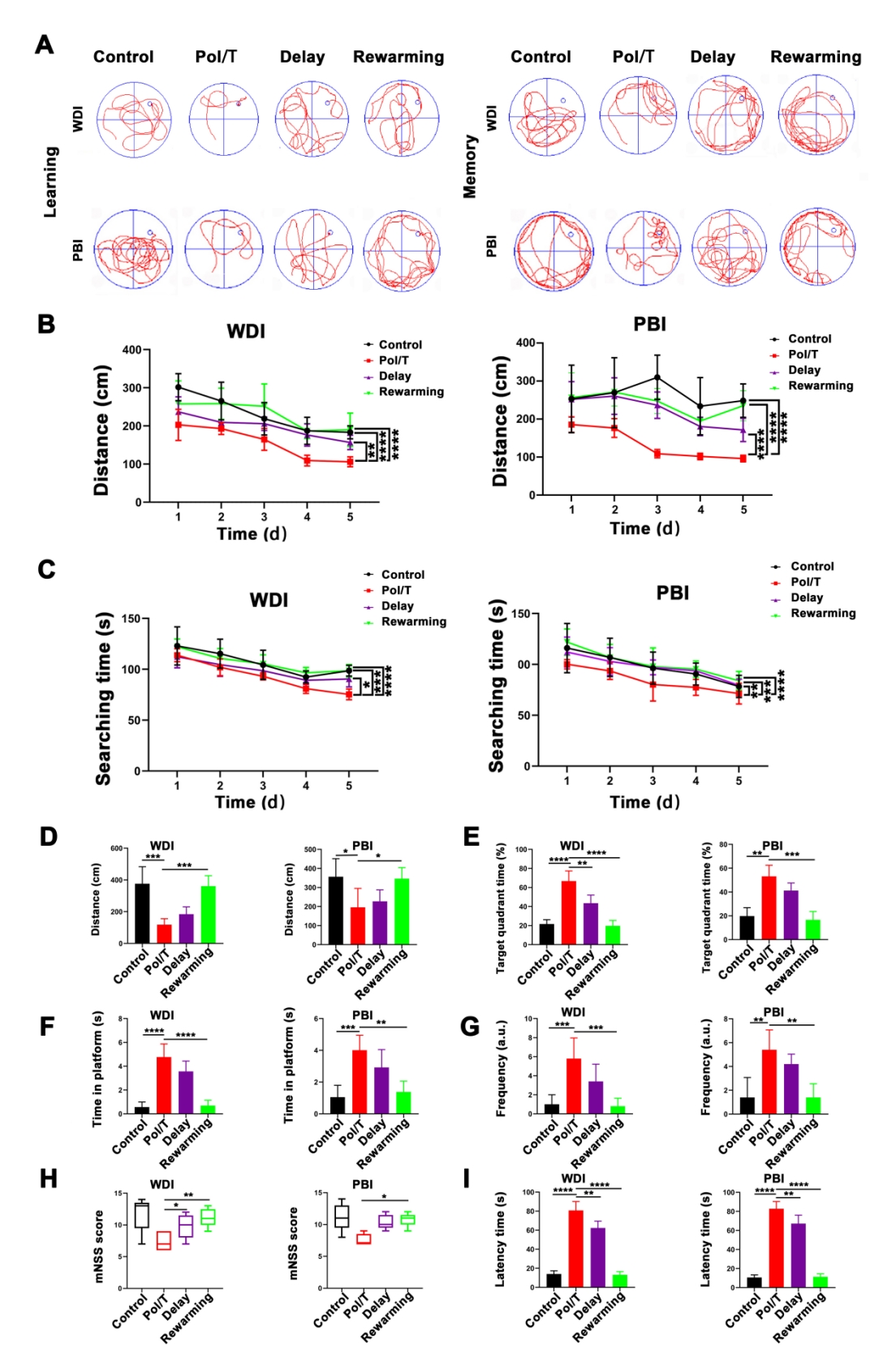


**Fig. S18** Functional recovery assessments by Morris water maze test, mNSS score and wire hanging test on 21 d after TBI. (A) Computer printouts of the swimming trajectories during the learning phase and memory phase. (B) Swimming distance to the platform and (C) searching time for the platform distance at the last trial each day during 5 d of training. (D) Swimming distance to the platform, (E) target quadrant time, (F) duration time on the platform and (G) frequency on the platform. (H) mNSS score was examined. (I) Motor function was evaluated by the wire hanging test. (B-G), (I) Data were presented as means ± SDs (n = 5 mice). Normality was checked using Shapiro-Wilk test. ^*^*P* < 0.05, ^**^*P* < 0.01, ^***^*P* < 0.001 and ^****^*P* < 0.0001 were performed using one-way ANOVA with Tukey’s post hoc test (consistent with normal distribution) and Kruskal-Wallis test (did not consistent with normal distribution). (H) Data are presented as median with range (n = 5 mice). ^*^*P* < 0.05 and ^**^*P* < 0.01 were performed using Kruskal-Wallis test.


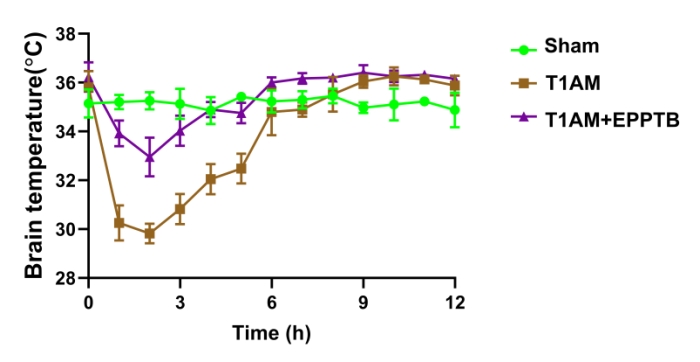


**Fig. S19** Brain temperature variation of Sham group, T1AM group and T1AM + EPPTB group. Data were presented as means ± SDs (n = 4 mice).
